# Hydrogels for Healing Radiation-Injured Tissues and Organs

**DOI:** 10.3390/gels12050450

**Published:** 2026-05-20

**Authors:** David Pawłowski, Kinga Słomska, Jakub Telszewski, Marcel Hubert Pilarski, Kamil Klimkowski, Julia Witkowska, Elżbieta Jankowska

**Affiliations:** Faculty of Chemistry, Department of Biomedical Chemistry, University of Gdansk, Wita Stwosza 63, 80-308 Gdansk, Poland; david.pawlowski@phdstud.ug.edu.pl (D.P.); kinga.slomska@phdstud.ug.edu.pl (K.S.); j.telszewski.881@studms.ug.edu.pl (J.T.); marcel.pilarski@phdstud.ug.edu.pl (M.H.P.); kamil.klimkowski@phdstud.ug.edu.pl (K.K.)

**Keywords:** biomaterial, hydrogel, radiotherapy, skin, mucosa, heart, lungs, bones, gastrointestinal tract

## Abstract

Radiotherapy remains one of the main pillars of cancer treatment and is used in more than half of all oncological patients. Despite continuous technological improvements, ionizing radiation inevitably causes damage to surrounding healthy tissues, leading to acute and chronic complications affecting multiple organs, including the skin, mucosa, heart, lungs, bones and gastrointestinal tract. Radiation-induced injuries significantly impair patients’ quality of life, limit therapeutic doses, and represent a major unmet clinical challenge. Hydrogels have emerged as promising biomaterials for managing radiation-induced damage due to their high content of water, tunable mechanics, and ability to mimic the extracellular matrix. Recent innovations have introduced functional systems, including stimuli-responsive, injectable, and bioactive hydrogels, capable of delivering antioxidants, growth factors, or living cells. Unlike traditional material-based reviews, this work proposes a novel classification framework based on the hydrogel’s mechanism of action within the pathophysiology of radiation injury. We evaluate how specific designs, such as ROS-scavenging matrices, barrier-forming injectable shields, and bioactive delivery vehicles, address distinct phases of inflammation and fibrosis. By providing a comprehensive overview of radiation-induced injuries across different organs, this review summarizes current hydrogel-based strategies for both prevention and therapy. We highlight the potential of these mechanistically aligned systems to protect healthy tissues, suppress chronic inflammation, and promote effective tissue regeneration.

## 1. Introduction

**Figure 1:**
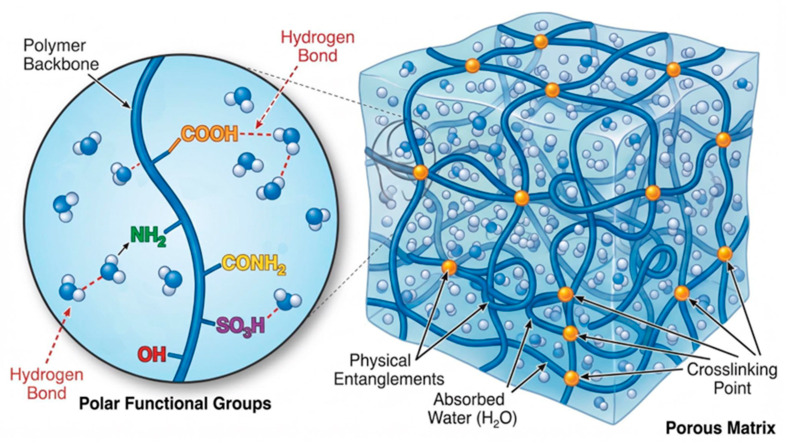
Schematic representation of a three-dimensional crosslinked hydrogel network showing polymer chains, crosslinking points, hydrophilic functional groups, water molecules, and hydrated pores. The figure was created with the Artlist AI tool (AI Suite 16.5K), informed by the data and descriptions from the publications cited in the chapter.

Hydrogels are three-dimensional crosslinked networks of hydrophilic polymers that can absorb and retain large amounts of water or biological fluids without dissolving, owing to the presence of polar functional groups such as –OH, –COOH, –NH_2_, –CONH_2_ and –SO_3_H along the polymer backbone [1]. These networks are formed by physical entanglements or chemical covalent bonds between polymer chains, resulting in a highly hydrated, porous matrix that closely mimics the extracellular matrix of native tissues (Figure 1) [2].

The foundational development of synthetic hydrogels dates back to the pioneering work of Wichterle and Lím, who introduced crosslinked poly(glycolmethacrylate) for soft-contact-lens applications [3]. Since then, hydrogels have evolved into versatile biomaterials with tunable physicochemical properties [4]. Their high equilibrium water content, often exceeding 90%, provides a moist healing environment essential for treating radiation-induced dermatitis and mucosal ulcers [5]. The ability to precisely modulate swelling ratios and mechanical moduli allows these materials to match the elasticity of various affected organs, from soft mucosal surfaces to more rigid lung or cardiac tissues, thereby minimizing mechanical irritation of already inflamed post-radiation sites [1]. Furthermore, the stimuli-responsive nature of smart hydrogels offers unique advantages in the context of radiotherapy. For instance, pH- or redox-responsive systems can be designed to trigger the release of radioprotective agents or antioxidants specifically within the acidic, oxidative environment of a radiation-induced inflammatory lesion [6]. Collectively, these properties transition hydrogels from simple wound dressings to sophisticated, bioactive platforms capable of addressing the complex, multi-organ pathophysiology of radiation-induced damage.

## 2. Advantages and Disadvantages of Hydrogels in Wound Treatment, with Specific Emphasis on Radiation Wounds

Hydrogels excel in maintaining a moist wound environment, which promotes keratinocyte migration, fibroblast proliferation, autolytic debridement, and re-epithelialization while preventing eschar formation. They are non-adherent, reduce pain during dressing changes, provide a cooling sensation, permit gas exchange, and absorb moderate exudate without causing periwound maceration [7,8] (Table 1).

Key disadvantages include limited absorbency in heavily exudative wounds, risking maceration, inferior mechanical strength relative to foam dressings, and higher cost than basic gauze [10]. Some hydrogels exhibit variable performance in moist desquamation phases or faster degradation under ionizing radiation exposure, with isolated reports of no benefit or slight delays in healing versus dry approaches in specific contexts [19,20]. Natural-based hydrogels, such as alginate and hyaluronic acid, often exhibit mechanical instability or reduced durability post-exposure to ionizing radiation due to radiation-induced chain scission, which compromises their structural integrity [21,22].

Overall, despite these limitations, the excellent biocompatibility of hydrogels, the ability to tailor their properties, and compelling evidence of their effectiveness make them the preferred method of wound treatment, emphasizing patient comfort and improved tissue regeneration. Many of these limitations can be effectively minimized through structural modifications, composite formulations, crosslinking optimizations, and incorporation of functional additives such as nanoparticles or bioactive agents [11,23].

## 3. Hydrogel Classification Based on Their Mechanism of Action in the Pathophysiology of Damage

**Figure 2:**
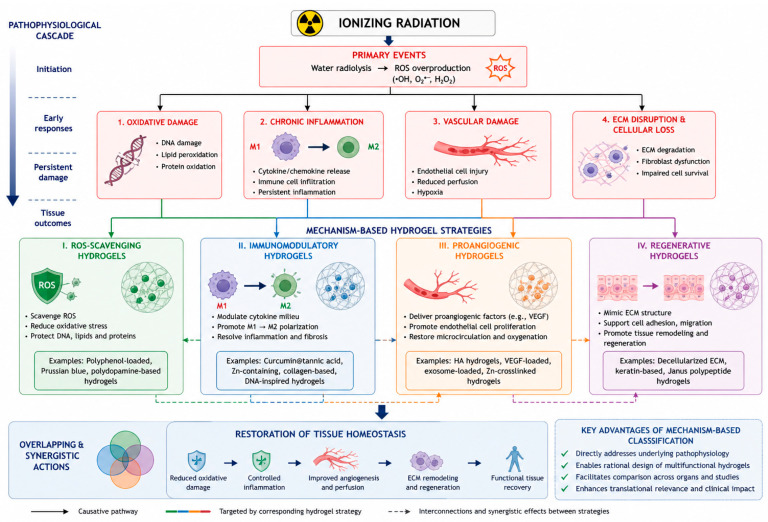
Schematic representation of the mechanism-based classification of hydrogels in radiation-induced tissue injury. Ionizing radiation triggers ROS overproduction, leading to oxidative damage, chronic inflammation, vascular impairment, and defective tissue remodeling. Corresponding hydrogel strategies are categorized as: (**I**) ROS-scavenging hydrogels interrupting oxidative stress, (**II**) immunomodulatory hydrogels restoring immune balance, (**III**) proangiogenic hydrogels promoting vascular regeneration, and (**IV**) regenerative hydrogels supporting extracellular matrix reconstruction and tissue repair. The overlap between categories reflects the multifunctional nature of advanced hydrogel systems. The figure was created with the Artlist AI tool (AI Suite 16.5K), informed by the data and descriptions from the publications cited in the chapter.

Hydrogels can be classified according to multiple criteria, reflecting their diverse physicochemical properties and synthesis strategies. Common categorizations include source of origin (natural, synthetic, or hybrid) [24], cross-linking mechanism (physical versus chemical) [25] and responsiveness to external stimuli (e.g., pH-sensitive, temperature-responsive, or redox-responsive) [1]. These frameworks are widely employed in comprehensive reviews of hydrogel design for biomedical applications, particularly in tissue engineering, drug delivery, and general wound healing, where emphasis is placed on material composition, mechanical tunability, and environmental adaptability. However, in the context of the present review focused on hydrogels for healing radiation-injured tissues and organs, we developed a hydrogel classification based on their mechanism of action. Traditional classifications of hydrogels provide valuable insights into material composition and physicochemical behavior, but they remain largely material-centric and do not directly reflect the biological processes underlying radiation-induced tissue damage. In contrast, the mechanism-based classification proposed in this review aligns directly with the key pathophysiological axes of radiation injury, including oxidative stress, chronic inflammation, vascular disruption, and impaired tissue remodeling (Figure 2, Table 2). This framework enables a more clinically relevant interpretation by linking hydrogel design to specific therapeutic targets and biological outcomes. Moreover, it facilitates rational design of multifunctional systems that simultaneously address multiple pathological pathways, which is particularly important in radiation-induced injuries characterized by overlapping and self-amplifying damage mechanisms.

### 3.1. Hydrogels Preventing Oxidative Stress

Ionizing radiation induces a rapid and sustained overproduction of reactive oxygen species (ROS) primarily through water radiolysis and secondary through mitochondrial dysfunction and protein misfolding, creating an overwhelming oxidative burden that exceeds endogenous antioxidant capacity [40,41,42,43]. This excess ROS directly inflicts oxidative damage on essential cellular components, including DNA (base modifications, single-strand breaks, and particularly lethal double-strand breaks), lipids (peroxidation leading to membrane destabilization), and proteins (carbonylation, fragmentation, and loss of function) [44,45,46,47,48]. Such macromolecular lesions disrupt genomic stability, mitochondrial energy metabolism, and cellular redox homeostasis, establishing a self-amplifying cycle of oxidative injury [41,42,49].

Persistently elevated ROS levels further activate redox-sensitive signaling cascades (e.g., MAPK pathways) and transcription factors, while upregulating pro-oxidant enzymes such as NADPH oxidase, lipoxygenases, cyclooxygenases, and nitric oxide synthase [47,49]. These events drive a chronic inflammatory response characterized by persistent cytokine release, immune cell recruitment, matrix metalloproteinase activation, extracellular matrix degradation, and fibroblast dysfunction [50,51,52]. The resulting microenvironment impedes timely re-epithelialization, collagen organization, and angiogenesis, ultimately manifesting as delayed wound closure, chronic ulceration, fibrosis, telangiectasia, and increased risk of carcinogenesis in severe or repeated exposures [43,51,53].

In response to the radiation-induced ROS → damage → inflammation axis, innovative hydrogel platforms have been engineered to intercept and neutralize excessive ROS directly at the injury site. These systems transition from passive dressings to active therapeutic interventions by incorporating ROS-responsive chemistries and antioxidant payloads, effectively modulating the local microenvironment to interrupt the cycle of chronic oxidative stress. Recent advancements in this field focus on several strategic approaches to ROS scavenging. One prominent strategy involves the integration of natural antioxidants, where hydrogel matrices, such as those based on carbomers or sodium alginate, are functionalized with polyphenols and natural acids, including ferulic acid or resveratrol [13,14]. These formulations effectively combine the physical benefits of a biocompatible matrix with the innate ability of the payload to suppress oxidative stress and neutralize free radicals. Parallel to this, more sophisticated catalytic systems have emerged, involving the embedding of nanoparticles with multi-enzyme mimetic activities directly into the hydrogel network. Examples such as Prussian blue nanoparticles or polydopamine exhibit peroxidase- and catalase-like properties, creating “smart” hydrogels that provide sustained, localized antioxidant defense superior to conventional symptomatic treatments [15]. Furthermore, the scope of ROS-responsive designs has expanded to include bioactive and cell-derived payloads. In these systems, hydrogels serve as carriers for biological mediators, such as ADSC-derived (adipose-derived stem cell) exosomes or specific functional proteins like IFI6. Such platforms do not merely scavenge ROS but also target downstream immunomodulatory pathways, effectively promoting a metabolic shift from a pro-inflammatory to a regenerative microenvironment [15,54].

### 3.2. Immunomodulatory Hydrogels

Radiation-induced wounds are characterized by chronic inflammation, driven by dysregulated cytokine profiles and persistent dominance of pro-inflammatory M1 macrophages, which hinder tissue regeneration and exacerbate fibrosis [55,56]. In normal wound healing, macrophages play a pivotal role, initially polarizing to the M1 phenotype to combat pathogens through secretion of reactive oxygen species and pro-inflammatory cytokines such as tumor necrosis factor α (TNF-α), interleukin 1β (IL-1β), IL-6, followed by a transition to the anti-inflammatory M2 phenotype that promotes resolution of inflammation, angiogenesis, and extracellular matrix remodeling [56,57,58,59]. However, in radiation burns, this balance is disrupted, with prolonged M1 activation leading to non-resolving inflammation, impaired phagocytosis, and sustained release of neurotoxic mediators like IFN-γ and FasL, perpetuating tissue damage [58,59,60,61]. Low-dose ionizing radiation has shown potential to modulate this by reducing pro-inflammatory cytokines (e.g., IL-1β, IL-6) and upregulating anti-inflammatory ones (e.g., IL-10, transforming growth factor-β1 (TGF-β), IL-4), facilitating M1-to-M2 shifts and enhancing neuroprotection [39]. Similarly, targeting pathways like Janus Kinase/Signal Transducer and Activator of Transcription (JAK/STAT) or Nuclear Factor kappa-light-chain-enhancer of activated B cells (NF-κB) can reprogram macrophages toward M2 dominance, inhibiting fibrosis and boosting natural killer cell activity against tumors [16,17,62].

**Figure 3:**
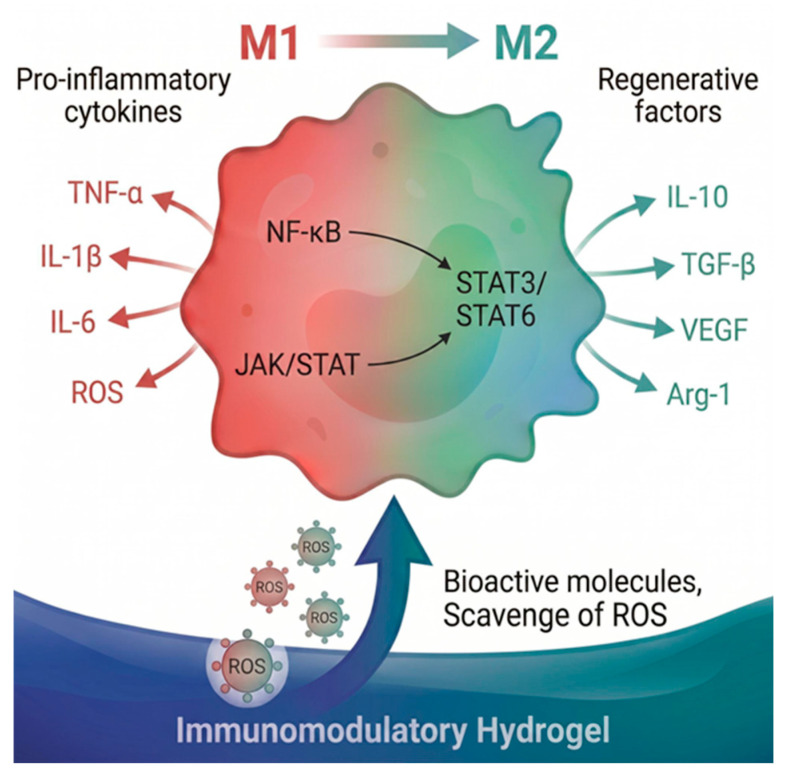
Hydrogel-induced macrophage polarization from pro-inflammatory M1 to pro-regenerative M2 phenotype. The immunomodulatory hydrogel releases bioactive molecules and scavenges ROS, inhibiting NF-κB and JAK/STAT pathways associated with M1 polarization while activating STAT3/STAT6 signaling to promote M2 markers (IL-10, TGF-β, VEGF, Arg-1). The figure was created with the Artlist AI tool (AI Suite 16.5K), informed by the data and descriptions from the publications cited in the chapter.

Immunomodulatory hydrogels offer innovative strategies to restore cytokine balance and macrophage polarization in radiation-damaged tissues by providing spatiotemporal control over inflammatory microenvironments [18]. These biomaterials mimic extracellular matrices, enabling sustained release of bioactive agents that recruit M1 macrophages to exert an antimicrobial effect, then promote their phenotypic switch to M2 for regeneration (Figure 3).

For instance, a curcumin@tannic acid nanoparticle-loaded gelatin methacryloyl hydrogel patch scavenges ROS chemically, shields against radiation physically via high water content, and drives M2 polarization, demonstrating enhanced repair in radiation dermatitis models through anti-inflammatory and adhesive properties [26]. An all-natural protocatechuic aldehyde-hybridized collagen hydrogel exhibits bioadhesive, antibacterial, and ROS-scavenging capabilities, directly converting M1 to M2 macrophages without external interventions, accelerating wound healing by shortening inflammation [27]. Temporal control is achieved in a zinc-hydroxyapatite nanoparticle-incorporated protein-alginate hydrogel, where early Ca^2+^ release enhances M1 activity for infection control, followed by sustained Zn^2+^ promoting M2-driven osteogenesis and angiogenesis [28].

Further innovations include DNA-inspired adenine-thymine paired self-healing hydrogels that activate STAT3/STAT6 pathways to polarize M1 to M2, fostering wound repair via dynamic Schiff base and hydrogen bonding for mechanical resilience [29]. Mechanoresponsive zwitterionic sulfobetaine methacrylate hydrogels with keratin-exfoliated MoS_2_ and phenytoin-loaded bee-wax nanoparticles respond to force by enhancing M2 polarization, proliferation, and antibacterial effects, addressing wounds through antifouling and adhesive features [30]. Catalyst-mediated acylhydrazone-crosslinked lysozyme-PEG hydrogels decouple viscoelasticity from equilibrium, activating JAK/STAT to suppress M1 markers (CD86, IL-1β) while upregulating M2 (CD204, VEGF) for improved healing [16]. Quercetin-solid lipid nanoparticle-embedded hyaluronic acid hydrogels inhibit M1 and elevate M2 polarization in vitro and in vivo, promoting osteogenesis by modulating inflammatory cytokines in bone immunomodulation [31]. These hydrogels innovatively integrate natural-derived components for biocompatibility, offering targeted immunomodulation to mitigate radiation-induced chronic inflammation and restore tissue homeostasis.

### 3.3. Proangiogenic Hydrogels

In the pathophysiology of radiation-induced tissue damage, ionizing radiation directly injures the vasculature, provoking endothelial cell detachment, apoptosis, and loss of key markers such as vascular endothelial cadherin and endothelial nitric oxide synthase (eNOS). These changes culminate in reduced capillary density, microvascular regression, and persistent ischemia that markedly delay regeneration and compromise wound healing [63,64,65]. Radiation further disrupts physiological angiogenesis by decreasing the expression of vascular endothelial growth factor (VEGF), angiopoietin-1, and Tie-2 while increasing angiopoietin-2 expression, collectively limiting endothelial proliferation and sprouting [66], while suppressing VEGFA and eNOS [64]. Compensatory responses such as elevated VEGF and HIF-1α expression may occur in normal tissue exposed to radiation, potentially accelerating repair, yet therapeutic regimens often reduce serum angiogenic cytokines and favor fibrosis [67,68,69]. Low-dose irradiation, however, can paradoxically promote neovascularization in ischemic limbs through mast-cell-derived VEGF release in a matrix metalloproteinase-9-dependent manner, underscoring VEGF’s pivotal role in orchestrating vascular regeneration [70]. Collectively, these processes highlight that angiogenesis constitutes the indispensable biological link between restored perfusion and successful tissue repair following radiation injury [71].

Targeted delivery of growth factors, above all VEGF, represents a cornerstone strategy to overcome radiation-induced vascular deficits. Hydrogels excel in this context as injectable or printable depots that provide localized, sustained VEGF presentation, function as tunable controlled-release systems, and replicate the extracellular matrix architecture to guide endothelial cell migration, tube formation, and neovessel maturation. Several innovative proangiogenic hydrogel platforms have been engineered to harness these properties. A composite hydrogel comprising human umbilical cord blood-derived mesenchymal stromal cells and porcine small intestinal submucosa promotes healing in radiation-wound models by amplifying angiogenic factor secretion from the stromal cells, particularly hepatocyte growth factor, which in turn recruits damaged endothelial cells and drives neovascularization [72]. A photo-crosslinked methacrylate hyaluronic acid hydrogel covalently functionalized with a prominin-1-binding peptide forms in situ under brief ultraviolet exposure. The tethered peptide markedly enhances VEGF recruitment, endothelial tubular formation, and cell migration in vitro, while in vivo studies in burn and excisional wounds demonstrate robust neovascularization and accelerated closure via VEGF-Akt pathway activation [32]. Cryogenically 3D-printed scaffolds integrating decellularized small intestinal submucosa, mesoporous bioactive glass, and exosome cargo enable prolonged exosome release that elevates local VEGF production, expands CD31-positive vessel area, augments blood perfusion, and stimulates angiogenesis, resulting in faster closure of full-thickness wounds [33]. Light-responsive 3D-printed hydrogel patches incorporating VEGF-decorated tetrapodal zinc oxide microparticles achieve on-demand VEGF release upon UV/visible-light activation while exerting antibacterial activity. These patches display low cytotoxicity, minimal immunogenicity, and improved wound closure in vivo [34]. A gelatin-methacrylate/dopamine-methacrylate hydrogel crosslinked with Zn^2+^ ions via metal coordination provides sustained zinc release together with potent antibacterial action. The composite accelerates infected wound resolution by enhancing vascularization, collagen deposition, and dermal regeneration [35]. Finally, a hierarchically structured DNA hydrogel (Agilegel) exploits covalent, base-pair and pore-level interactions to achieve sequential release of VEGF-α, silver nanoclusters, and IL-10. The initial VEGF-α wave directly stimulates endothelial proliferation, angiogenesis, and extracellular matrix assembly, thereby promoting rapid neovascularization and closure of wounds while simultaneously addressing infection and oxidative stress [36].

### 3.4. Hydrogels Promoting Regeneration and Remodeling

Biomimetic hydrogels are engineered to replicate the native extracellular matrix (ECM) architecture and mechanics, facilitating integrin-mediated focal adhesions that are essential for guiding cell migration and mechanotransduction. These matrices offer a compliant, porous microenvironment that helps restore cytoskeletal dynamics and paxillin-rich adhesions, which are typically impaired in irradiated cells [9]. Beyond structural support, regenerative hydrogels are designed to modulate the immune microenvironment, specifically promoting macrophage polarization toward pro-regenerative M2 phenotypes, a transition crucial for the secretion of factors that support angiogenesis and physiological collagen remodeling [73].

Within this therapeutic framework, several specialized strategies have emerged to enhance the regenerative potential of these scaffolds. One prominent approach involves the development of ECM-derived systems, where hydrogels incorporating components like decellularized dermal matrices, keratin, or fibronectin directly supply missing matrix proteins and growth-factor binding sites to the damaged tissue [37]. This structural mimicry is often complemented by the use of functionalized dynamic carriers, where the incorporation of cells or cell-derived exosomes adds a layer of paracrine signaling. These carriers significantly prolong the retention of pro-regenerative cargo, such as miR-221-3p, at the injury site, thereby sustaining the delivery of signals that enhance fibroblast migration and keratinocyte epithelialization [70,71].

Furthermore, the field has seen the rise of innovative multifunctional synthetic designs, including self-assembling heparin-mimetic peptides and gene-engineered Janus polypeptides. The platforms integrate ECM mimicry with advanced features such as ROS scavenging, on-demand oxygenation, and mechanical tension relief to further optimize the biological performance of the graft [39]. By integrating these diverse features, regenerative hydrogels effectively address both the structural deficits and the dysregulated cellular crosstalk characteristic of damaged tissues, orchestrating a comprehensive transition from chronic inflammation to orchestrated tissue repair. Building on these mechanistic foundations, the subsequent sections review the therapeutic application of hydrogels in radiation-induced injuries across various tissues and organ systems. We first focus on cutaneous and mucosal damage, which represent the most common and clinically visible manifestations of radiation toxicity, and then discuss hydrogel-based approaches developed for the internal organs commonly compromised during radiotherapy, namely the gastrointestinal tract, lungs, heart, and bone.

## 4. Hydrogel-Based Strategies in the Treatment of Radiation-Induced Injuries Across Different Tissues and Organ Systems

### 4.1. Skin

Despite its many advantages, radiotherapy remains associated with significant adverse effects on healthy tissues, particularly the skin, which is highly susceptible to ionizing radiation-induced damage due to its constant renewal and the presence of rapidly proliferating and differentiating cells [74]. As radiotherapy constitutes a cornerstone of oncological treatment and is used in approximately 50–60% of cancer patients [75], radiation-induced skin injuries (RISI) are among the most common complications observed both during and after therapy [23]. Skin reactions affect nearly 85% of patients undergoing radiotherapy and may range in severity from mild erythema and desquamation to severe ulceration [76].

**Figure 4:**
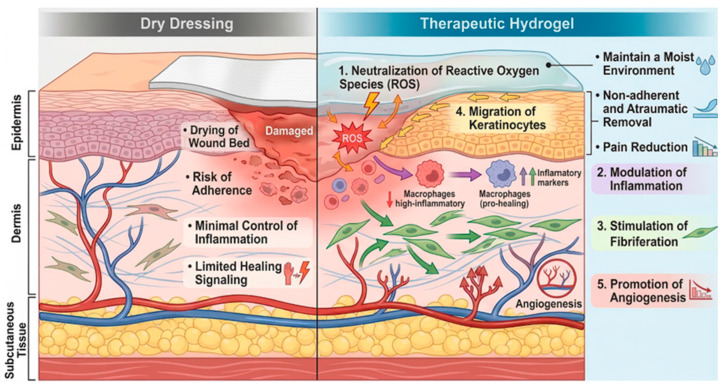
Schematic representation of hydrogel action in a post-radiotherapy wound. The illustration shows a cross-section of skin with hydrogel applied on the wound. The figure was created with the Artlist AI tool (AI Suite 16.5K), informed by the data and descriptions from the publications cited in the chapter.

Hydrogels developed for the treatment of radiation-induced skin injury can be broadly classified according to their predominant therapeutic strategy. Most systems are designed to suppress oxidative stress, modulate inflammatory and immune responses, promote extracellular matrix reconstruction and tissue regeneration [23,77]. Multifunctional hydrogel formulations demonstrate accelerated closure, higher healing rates, reduced pain, and lower infection risk compared to conventional dry dressings in radiation-damaged tissues [22] (Figure 4).

Because excessive generation of reactive oxygen species (ROS) represents one of the earliest and most critical events in radiation-induced tissue damage, antioxidant-based hydrogels have emerged as the most extensively studied group [78]. Consequently, therapeutic approaches based on the initial elimination of ROS followed by stimulation of tissue repair are considered particularly important in the management of radiation-induced skin wounds. Effective treatment therefore requires not only enhancing the antioxidant capacity of the skin but also preventing cellular senescence and further tissue deterioration [79]. Among antioxidant and ROS-scavenging systems, a carbomer-based ferulic acid (FA) hydrogel markedly accelerates RISI recovery by suppressing oxidative stress, reducing inflammation, and inactivating the NLRP3 inflammasome at both in vitro and in vivo levels, where multiple FA concentrations promote collagen deposition, tissue reconstruction, and normalization of skin blood flow [14]. Similarly, dual-network photocrosslinkable hydrogels (PHF@Res) embedding polyvinylpyrollidone-modified Prussian blue nanoparticles and resveratrol exhibit broad free-radical scavenging together with peroxidase- and catalase-mimetic activities to support fibroblast migration and promote limb regeneration [13]. The use of polyphenols like epigallocatechin gallate (EGCG) is also crucial, as this compound removes superoxide anions, hydroxyl radicals, and hydrogen peroxide while protecting DNA and inhibiting the proteasome, a key regulator of inflammation controlling cytokines such as IL-1β, IL-6, IL-8, and TNFα [80,81]. To overcome challenges of direct spraying, a hydrogel composed of chitosan and gelatin grafted with photosensitive methacrylic anhydride was developed to deliver EGCG via minimally invasive injection, enhancing the expression of genes related to angiogenesis [82]. Tannic acid is another antioxidant used in hydrogels containing *N*-acryloyl glycinamide and *N*-hydroxyethyl acrylamide, which exhibit self-healing capabilities and mechanical stability, often incorporating live *Lactobacillus reuteri* strains to respond to the wound microenvironment [78,83]. Other antioxidant formulations include caffeoyl chitosan and boronic acid-grafted gelatin methacrylate, which strengthen the expression of CD31 [84], as well as interpenetrating networks made of gelatin grafted with dopamine and curcumin-containing nanoparticles for real-time drug tracking [11]. Flavonoids such as baicalin, delivered via temperature-sensitive liposome systems, and dihydromyricetin nanocapsules demonstrate efficacy in protecting against DNA damage [85,86,87]. Similarly, topical gels containing hesperetin exhibit potent antioxidant and anti-inflammatory properties by combating oxidative stress, preventing damage to vital skin structures such as DNA, lipids, and proteins, and inhibiting inflammatory reactions through suppression of cytokines and enzymes [88]. Finally, phycocyanin-based copper sulfide nanoparticles encapsulated in alginate microspheres not only exhibit ROS-scavenging activity but also act as antibacterial agents and accelerate epidermal regeneration [89].

A second important group comprises immunomodulatory hydrogels designed to attenuate chronic inflammation and restore immune homeostasis in irradiated tissues. An IFI6-functionalized hydrogel incorporating polydopamine and sodium alginate combines excellent ROS scavenging, bioadhesion, and antibacterial properties with targeted activation of the SSBP1/HSF1 signaling axis, thereby reducing ROS accumulation, improving the immune microenvironment, and stimulating fibroblast proliferation and vascularization [15]. Likewise, adipose-derived stem cell exosomes delivered via biocompatible carriers alleviate RISI by attenuating oxidative stress, modulating macrophage polarization, and inhibiting apoptosis or pyroptosis [54]. The therapeutic potential of regenerative hydrogels is further evidenced by acellular dermal matrix hydrogels prepared from porcine dermis, which significantly accelerate healing by decreasing wound area and radiation-injury scores, increasing epithelial thickness and hair-follicle regeneration, and shifting macrophages toward IL-10-high M2 phenotypes while downregulating IL-1β and IL-6 [90]. Human-adipose-ECM hydrogels similarly alleviate late fibrosis by driving endothelial cell-mediated M2 polarization [91].

Another major category includes regenerative and ECM-mimicking hydrogels that directly support tissue reconstruction and restoration of skin architecture. Decellularized porcine dermal hydrogels rescue radiation-induced capsule fibrosis, normalize tissue architecture, and support adipogenesis without enhancing unwanted fibroblast infiltration [92]. Keratin-based hydrogels derived from hair promote post-radiation wound closure by enhancing fibroblast proliferation and ordered collagen deposition, while topical fibronectin augments angiogenesis and reduces inflammatory infiltrate when delivered in supportive matrices [93,94]. Collectively, these biomimetic systems promote collagen remodeling, epithelial regeneration, vascularization, and restoration of dermal appendages in irradiated skin.

Recent advances increasingly focus on multifunctional smart hydrogels integrating antioxidant, antibacterial, oxygenating, and regenerative properties within a single platform. Advanced multifunctional systems such as the gene-engineered Janus polypeptide hydrogel combine bacterial-clearance and on-demand oxygenation modules with RGD motifs that relieve mechanical tension, yielding near-complete (98.83%) wound closure by day 21 and an eight-fold increase in dermal appendages [95]. Self-healing and stimuli-responsive systems further enhance adaptability to the wound microenvironment and improve therapeutic precision [78,83]. Collectively, these interventions counteract hypoxia-driven myofibroblast persistence and TGF-β1/Smad-mediated ECM overproduction, thereby restoring the regenerative cascade in radiation-damaged skin [96,97].

### 4.2. Mucosa

Radiation-induced oral mucositis (RIOM) represents one of the most debilitating complications in patients undergoing radiotherapy for head and neck cancers. The condition arises from the direct DNA damage of basal epithelial cells and the subsequent generation of reactive oxygen species, which trigger complex inflammatory cascades involving NF-κB, TNF-α, and various interleukins [98]. This process leads to mucosal atrophy, ulceration, and severe pain, often necessitating treatment interruptions and compromising the patient’s nutritional status. A significant therapeutic challenge in managing RIOM lies in overcoming the suboptimal retention of drug delivery systems within the highly dynamic and moist environment of the oral cavity [99]. Continuous saliva secretion and mechanical movements of tissues associated with speaking, chewing, and swallowing lead to rapid washout of conventional formulations, such as rinses or sprays, preventing the maintenance of therapeutic concentrations of active substances at the ulcer site. The success of any mucosal intervention therefore begins with achieving high wet adhesion to ensure stable binding to the mucin layer. For instance, positively charged natural polymers, such as chitosan (often cross-linked with genipin to improve bioavailability), demonstrate the ability to interact strongly with negatively charged sialic acid residues in mucin [100]. This electrostatic matching significantly extends the residence time of the therapeutic load. Similar effectiveness is seen in hydrogels functionalized with catechol groups, inspired by the chemistry of marine organisms [101]. These groups mediate strong mucosal tissue binding through mechanisms such as hydrogen bonding, electrostatic interactions, Michael addition reactions, or Schiff base formation [102,103]. Other innovative approach is the EPBA@PC-HD hydrogel, which achieves stable mucosal adhesion due to the rearrangement of cholesterol micelles and displacement of water from the tissue interface [102].

Radiotherapy not only damages the body’s physical barriers, such as skin and mucosal tissues, but also induces systemic immunosuppression, which makes these tissues highly susceptible to bacterial infections [11,12,103]. These infections, particularly those caused by drug-resistant pathogens such as *Pseudomonas aeruginosa* or methicillin-resistant *Staphylococcus aureus* (MRSA), significantly exacerbate inflammation, delay healing processes, and can lead to deep tissue necrosis [103]. Consequently, effective infection control is one of the greatest challenges in the clinical management of radiation-induced wounds and oral mucositis. Although hydrogels represent a promising platform for supporting tissue regeneration, traditional dressings of this type often lack sufficient antimicrobial properties, which in some cases could promote the formation of protective bacterial biofilms on the wound surface, preventing antibiotic penetration [11]. To overcome this limitation, contemporary research focuses on designing multifunctional hydrogels that actively combat bacterial colonization by incorporating specific antibacterial agents or modifying the hydrogel matrix itself. One leading approach involves local delivery of broad-spectrum antibiotics, such as minocycline hydrochloride, embedded within hydrogels responsive to wound-environment stimuli [12]. In the context of oral mucositis treatment, it has been shown that hydrogels releasing minocycline in response to elevated ROS levels are highly effective at inhibiting the growth of both Gram-positive and Gram-negative bacteria, including *Escherichia coli* and MRSA [12]. This intelligent, sustained drug release prevents secondary infections and synergistically mitigates inflammatory responses, actively supporting the regeneration of damaged tissues and breaking the vicious cycle of oxidative stress and inflammation. Concurrently, researchers are exploring the use of natural plant extracts, such as baicalin, which exhibit strong antibacterial activity against drug-resistant strains while presenting a low risk of inducing microbial resistance [103]. Integration of baicalin with positively charged polymeric hydrogel matrices generates a synergistic bactericidal effect, where cationic polymer segments disrupt the negatively charged bacterial cell membranes, drastically reducing bacterial populations within irradiated wounds [103]. Furthermore, innovative strategies include the use of materials with inherent antibacterial properties, such as chitosan or graphene oxide polymers, as well as incorporation of metal nanoparticles, significantly expanding the repertoire of modern infection-resistant dressings [11,100,103].

Multifunctional hydrogels can actively participate in therapy also by scavenging ROS and enabling targeted drug release [12]. An example of an advanced solution is the QTMP-Gel, based on the dynamic crosslinking of quaternized chitosan with tannic acid. This hydrogel exhibits strong adhesion to moist mucosal tissues, resulting from the formation of numerous hydrogen bonds and dynamic interactions between cationic polymer chains and the negatively charged mucosal surface [12,104]. The material exploits the oxidation sensitivity of the catechol structures in tannic acid, allowing controlled degradation of the polymer network in the presence of high ROS concentrations and intelligent release of active substances directly at the site of inflammation. In the context of regenerating deeper injuries, studies highlight the benefits of hierarchical therapeutic strategies that combine immediate ROS scavenging by tannic acid with subsequent release of bioactive probiotics, such as *Lactobacillus reuteri* [78]. This system, designated Gel/LT, exploits the pH sensitivity of a polyphenol-metal coating in the wound bed, enabling sequential action: first, elimination of more than 90% of free radicals and inhibition of tissue necrosis, followed by stimulation of angiogenesis and collagen regeneration [78]. To the ROS-scavenging systems belongs biomimetic oCP@As hydrogel, which stimulate RAD51 protein expression, directly supporting the repair of DNA double-strand breaks [105]. The hydrogel forms an ECM-like nanofiber structure and contains oxidized chondroitin sulfate, which modulates inflammatory responses by adsorbing pro-inflammatory factors. Animal studies demonstrated that oCP@As promotes epidermal regeneration and angiogenesis and reduces pro-inflammatory cytokine expression [105]. The effectiveness of such systems can be enhanced by incorporating components with catalytic and anti-inflammatory properties. Platinum nanoparticles embedded in the hydrogel matrix exhibit enzyme-mimicking activity, similar to natural enzymes such as superoxide dismutase and catalase, enabling continuous decomposition of harmful superoxide anions and hydrogen peroxide into safe water and oxygen [12].

Complementing these antioxidative and anti-inflammatory strategies are hydrogels designed primarily to promote extracellular matrix reconstruction and epithelial regeneration. Composite G-PVA hydrogels containing core–shell microgels enable simultaneous delivery of lidocaine for pain relief and epidermal growth factor to stimulate tissue repair [106]. The ability to simultaneously relieve pain and stimulate epithelial regeneration is also realized in composite hydrogels based on hyaluronic acid and polyvinyl alcohol, where the incorporation of core–shell microgels allows spatial and temporal separation of lidocaine and growth factor release [101,106]. Aggregated research results indicate that such designed biomaterial platforms significantly accelerate regeneration, reducing the ulcerated area by more than half [104].

In advanced stages of damage where fibrosis dominates, a key strategy is the use of mesenchymal stem cells (MSCs) embedded in hydrogel scaffolds. Materials such as hyaluronic acid or silanized hydroxypropyl methylcellulose isolate MSCs from the aggressive inflammatory environment, providing them with conditions for survival and the secretion of growth factors [107,108,109]. In the context of radiation-induced mucosal damage (including oral mucositis, esophageal injury, and intestinal or colorectal damage) MSCs encapsulated in hydrogels have proven highly effective by improving cell survival, retention, paracrine activity, and targeted delivery [110]. A fundamental mechanism behind this efficacy is the modulation of the immune microenvironment. Several studies directly demonstrate that MSCs delivered in hydrogels inhibit pro-inflammatory M1 macrophages (CD68 markers) and promote reparative M2 phenotypes [109,111]. Specifically, in vivo evidence shows that hydrogel-encapsulated MSCs reduce the presence of M1 macrophages while increasing the population of M2 macrophages [111]. For instance, the use of YIGSR/RGD hydrogels in combination with MSCs has been shown to significantly increase the infiltration of CD206 macrophages while simultaneously decreasing CD68 infiltration [109,112]. Due to the appropriate porosity and bioactive properties of the scaffolds, these cells further stimulate angiogenesis and the regeneration of basal epithelial cells. Ultimately, these synergistic effects result in the restoration of organ functionality and the prevention of irreversible tissue scarring [108,109].

### 4.3. Gastrointestinal Tract

Irradiation of abdomino-pelvic malignancies can result in both acute and chronic damage to organs of the gastrointestinal tract. These injuries arise from direct interactions of ionizing radiation with cellular macromolecules, as well as indirect effects mediated by reactive oxygen species, leading to DNA and RNA damage, altered gene expression, protein modification, cellular senescence, and genomic instability [49,113,114]. The gastrointestinal tract is one of the most radiosensitive organs. This high sensitivity arises from the rapid turnover of epithelial stem cells located at the base of intestinal crypts. Reactive oxygen species generated by ionizing radiation trigger crypt cell apoptosis and compromise epithelial barrier integrity, resulting in increased mucosal permeability, nutrient and fluid loss, and bacterial translocation, which collectively amplify local inflammation [115]. The persistent inflammatory milieu, together with stem cell depletion and ischemic conditions, ultimately impairs tissue repair and may lead to chronic pathological outcomes such as ulceration, fibrosis, or fistula formation [116].

One promising approach to reducing radiation toxicity is the physical separation of target tissues from adjacent organs at risk. Hydrogels have emerged as particularly attractive materials for this purpose. Due to their biocompatibility and tissue-like radiological properties, hydrogels can absorb radiation in a manner similar to normal tissues. When implanted between the tumor and radiosensitive structures, they effectively reduce the radiation dose delivered to healthy organs. Experimental and clinical studies have demonstrated that hydrogel spacers can decrease radiation exposure to salivary glands in head and neck cancer [117,118], to the rectum in prostate cancer [119], and to the duodenum in pancreatic cancer [120], with associated improvements in gastrointestinal symptoms following radiotherapy [121,122]. By reducing radiation exposure to organs at risk, they enable dose escalation, hypofractionation, and potentially better tumor control.

The most extensive clinical experience with hydrogel spacers has been gained in prostate cancer. Since its approval by the US Food and Drug Administration, the SpaceOAR system has been widely adopted in radiotherapy protocols. This biodegradable polyethylene glycol (PEG) hydrogel creates a temporary space between the prostate and rectum, thereby reducing radiation exposure to the anterior rectal wall [123]. Clinical studies have shown that hydrogel implantation can achieve several millimeters of separation, leading to significant reductions in rectal dose parameters and lower rates of acute and late gastrointestinal toxicity [124,125,126,127]. These dosimetric benefits have been observed across different radiotherapy modalities, including external beam radiotherapy, stereotactic body radiotherapy (SBRT), and brachytherapy. In prostate brachytherapy, both hydrogel and balloon spacers have been reported to reduce rectal dose metrics by approximately 15–50%, accompanied by a marked decrease in clinically significant gastrointestinal toxicity. Similar dose-sparing effects have been documented in gynecological cancers, where spacer balloons or injectable gels reduced radiation exposure to the bladder and rectum without compromising tumor coverage. Overall, spacer use has been associated with fewer late complications and improved patient-reported quality of life [128]. In the context of high-dose SBRT, hydrogel spacer placement significantly reduced the incidence of rectal ulcers [129]. Among the biomaterials evaluated for this purpose, PEG-based hydrogels have gained preference over earlier materials such as hyaluronic acid or collagen, as PEG hydrogels tend to maintain their volume and structural integrity for longer periods under irradiation [130,131,132]. Hydrogel-based spacing has also been explored in pancreatic cancer, where gastrointestinal toxicity remains a major limiting factor for dose escalation. It has been demonstrated that endoscopic ultrasound-guided injection of PEG hydrogel is feasible and increases the distance between the pancreatic head and the duodenum. The hydrogel is clearly visible on imaging and undergoes gradual resorption within a few months, providing a suitable temporal window for radiotherapy [133]. Similarly, computed tomography-guided or endoscopic hydrodissection techniques have been proposed to allow higher radiation doses in pancreatic adenocarcinoma by physically separating the tumor from adjacent gastrointestinal structures, potentially improving treatment outcomes without the need for surgically implanted spacers [134]. Despite their advantages, traditional preshaped hydrogels often require surgical implantation, whereas injectable hydrogels offer minimally invasive delivery but may carry risks such as inflammation or material displacement [135]. To address these limitations, ongoing research focuses on improving hydrogel formulations by incorporating anti-inflammatory agents or using alternative cross-linking strategies. Examples include photo-cross-linkable hydrogels that modulate hypoxia-related pathways [136], drug-loaded hydrogels for targeted anti-inflammatory therapy [137,138,139,140], and multimodal injectable hydrogels with enhanced imaging visibility [141].

While hydrogel spacers primarily function as physical barriers to reduce radiation-induced damage, their clinical implementation does not fully eliminate late tissue toxicity, particularly in radiosensitive organs such as the gastrointestinal tract. Thus, beyond strategies aimed at dose optimization and organ protection, there remains a critical need for therapeutic approaches capable of actively promoting tissue repair and regeneration after radiation injury.

In this regenerative context, hydrogels have gained increasing attention as bioactive scaffolding platforms designed to support localized cell delivery and tissue reconstruction. Beyond serving as passive matrices, they may actively modulate the post-irradiation niche by enhancing cell retention, protecting transplanted cells from inflammatory stress, and enabling sustained paracrine signaling. Such properties are particularly relevant for cell-based regenerative strategies, including those employing mesenchymal stromal cells [142]. Hydrogels can serve as supportive platforms for localized cell delivery as their high-water content and structural similarity to the extracellular matrix allow them to provide a permissive microenvironment for cell survival and function [143]. Moreover, their injectability and in situ crosslinking properties enable minimally invasive administration and spatially controlled delivery at the site of injury. A notable example is the silanized hydroxypropylmethyl cellulose (Si-HPMC) hydrogel, developed as an injectable scaffold for MSC encapsulation and colonoscopic delivery [107]. In a rat model of radiation-induced colonic injury, local administration of adipose-derived MSCs embedded in Si-HPMC resulted in improved epithelial architecture and reduced hyperpermeability compared to systemic or non-encapsulated cell delivery. Nevertheless, even when delivered locally, MSC survival remains challenged by the hostile post-irradiation microenvironment characterized by hypoxia, oxidative stress, and persistent inflammation. To further enhance therapeutic outcomes, combined approaches integrating matrix-mimetic molecules have been proposed. In this context, Moussa et al. demonstrated that conditioning the injured tissue with heparan sulfate mimetics, aimed at restoring extracellular matrix organization and growth factor signaling, in combination with hydrogel-protected MSC delivery, significantly improved tissue regeneration in two relevant animal models [144]. This strategy led to a marked reduction in injury scores and promoted epithelial repair, supporting the concept that engineered biomaterial niches can actively modulate the regenerative microenvironment and potentiate cell-based therapies.

Beyond cell therapy, hydrogels have also been explored as vehicles for localized delivery of bioactive compounds to mitigate radiation-induced gastrointestinal toxicity. Radiation-induced proctitis (RIP), a common adverse effect in patients treated for pelvic cancers, exemplifies a condition where local inflammation, oxidative stress, and impaired mucosal healing converge. Semi-synthetic glycosaminoglycans derived from hyaluronic acid exhibit anti-inflammatory and tissue-protective properties but suffer from limited tissue penetration. To address this, Jensen et al. developed an in situ gelling rectal delivery system based on silk-elastin-like protein polymers, enabling sustained release and enhanced local accumulation of glycosaminoglycans in rectal tissue [145]. This system simultaneously targeted multiple pathological mechanisms underlying RIP, including reactive oxygen species scavenging, cytokine modulation, and epithelial regeneration, illustrating the therapeutic versatility of hydrogel-based platforms.

Polysaccharide-based hydrogels represent another class of biomaterials with significant translational potential due to their intrinsic biocompatibility, biodegradability, and mucoadhesive properties. Chemically modified Moringa oleifera gum, for example, has been combined with chitosan or synthetic polymers to produce hydrogel systems exhibiting prolonged mucosal retention and sustained release behavior [146,147]. Radiation-induced grafting of vinyl monomers onto moringa gum further yielded hydrogels characterized by antioxidant activity and controlled release profiles without an initial burst effect [148]. Although originally developed for conventional gastrointestinal drug delivery, such properties suggest that these materials could also serve as adaptable carriers for therapeutic agents aimed at alleviating radiation-induced intestinal damage.

More advanced regenerative strategies have employed synthetic hydrogels as delivery vehicles for complex biological constructs. Cruz-Acuña et al. demonstrated that an engineered PEG-based hydrogel (PEG-4MAL) could effectively deliver human intestinal organoids to colonic wounds, significantly enhancing mucosal repair compared to organoids or hydrogel alone [149]. This work provided proof-of-concept that synthetic hydrogels can support localized engraftment of tissue-engineered constructs and promote functional tissue regeneration in vivo.

**Figure 5:**
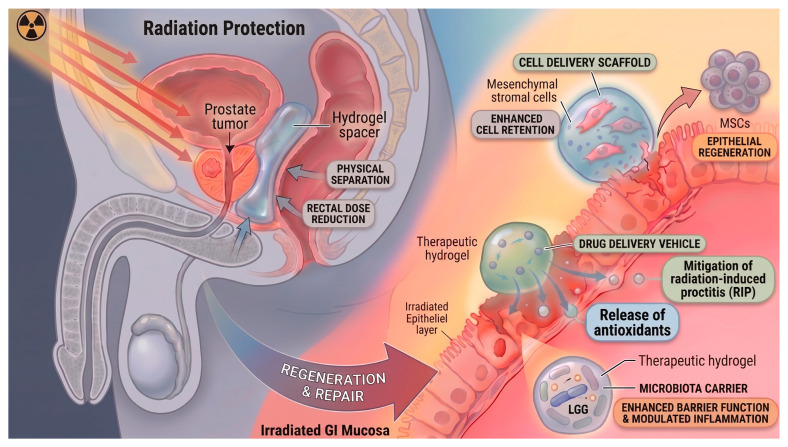
Hydrogel-based strategies for mitigating radiation-induced gastrointestinal damage. The schematic summarizes the dual approach of hydrogel technology: radiation protection via anatomical displacement (spacer hydrogels) and therapeutic intervention via advanced delivery systems. These systems facilitate the delivery of MSCs, bioactive molecules, and probiotics to repair the irradiated epithelial layer and mitigate the symptoms of radiation-induced proctitis. The figure was created with the Artlist AI tool (AI Suite 16.5K), informed by the data and descriptions from the publications cited in the chapter.

Hydrogel platforms have also been applied to microbiota-based interventions. Fecal microbiota transplantation and probiotic therapies have shown potential in mitigating radiation-induced bowel injury, yet their clinical application is limited by poor microbial viability and safety concerns. Gu et al. addressed these challenges by encapsulating Lactobacillus rhamnosus GG in a chitosan-based hydrogel system, which protected bacterial viability and enhanced epithelial barrier function while reducing inflammatory cytokine levels in irradiated models [150]. This approach underscores the capacity of hydrogel systems to act not only as passive carriers, but also as active modulators of the intestinal microenvironment (Figure 5).

Finally, recent developments in injectable and stimuli-responsive hydrogels further expand their therapeutic relevance. Next-generation injectable systems enable minimally invasive delivery and can be engineered to incorporate anti-inflammatory or bioactive components [135]. Examples include photo-crosslinkable hydrogels that restore hypoxia-related signaling pathways [136], pectin-based systems for colon-targeted drug delivery [137], and tannic acid-crosslinked hydrogels with intrinsic anti-inflammatory properties [138]. Collectively, these advances highlight the growing potential of engineered hydrogel systems as multifunctional platforms for the localized treatment of radiation-induced gastrointestinal injury [113].

### 4.4. Lungs

Radiation-induced lung injury (RILI) results from a cascade of damage initiated by ionizing radiation in alveolar and endothelial cells, leading to oxidative stress, inflammation, and fibrotic remodeling of the lung. RILI is the most common non-malignant complication of radiation therapy for thoracic malignancies. Radiation-induced lung injury (RILI) results from a cascade of damage initiated by ionizing radiation in alveolar and endothelial cells, leading to oxidative stress, inflammation, and fibrotic remodeling of the lung. RILI is the most common non-malignant complication of radiation therapy for thoracic malignancies. According to the latest GLOBOCAN estimates, reported in 2024, there were approximately 20 million new cancer cases worldwide in 2022, with lung and female breast cancer representing the two most common malignancies and oesophageal cancer ranking among the top causes of cancer death [151]. Using these incidence data together with published radiotherapy utilization rates, we estimate that over 3.1 million patients per year undergo thoracic radiotherapy for lung, breast, or oesophageal cancer [152]. The estimated incidence of RILI varies widely across studies, likely due to varying definitions of clinically significant lung injury, however drawn from recent reviews and disease-specific cohorts about 30% of these patients (approximately 0.93 million) develop RILI [153]. RILI is a major dose-limiting factor in the treatment of thoracic malignancies. To reduce the risk and severity of RILI, the treatment plans are modified by narrowing the irradiation field or lowering the radiation dose. However, this could adversely affect local tumor control rates. The prognosis of patients with RILI is often poor, with a median survival time of less than 3 years [154]. Therefore, developing new strategies to prevent, delay, or halt the development of RILI is essential to improve tumor control, overall survival, and maintain the quality of life of patients receiving thoracic radiotherapy.

The pathology of RILI was originally described in 1925 by Evans who classified acute RILI as radiation pneumonitis and chronic RILI as radiation pulmonary fibrosis [155]. The early, acute phase typically occurs within the first six months after radiotherapy and is characterized by an inflammatory response in the pulmonary tissue. The late phase generally develops beyond six months post-irradiation and is defined by irreversible fibrotic remodeling, excessive extracellular matrix deposition, and permanent scarring of the lung parenchyma. Radiotherapy damages normal cells by both direct and indirect means. As a direct effect of ionizing radiation on lung tissue, nuclear and mitochondrial DNA damage and the generation of reactive oxygen and nitrogen species are described [156]. Such damage initiates multidimensional DNA damage responses within hours and leads mainly to a transient arrest of the cell cycle [157]. In case of low damage, the transient cell cycle arrest allows for DNA repair and cell recovery. However, if damage is too significant for repair, cells might undergo permanent cell cycle arrest and enter a senescent state or undergo acute or delayed forms of cell death. ROS are generated in various lung cell types, including endothelial cells, neutrophils, eosinophils, alveolar macrophages, and alveolar epithelial cells [158]. Excessive ROS production results in oxidative stress, which damages endothelial cells, disrupts the integrity of intercellular junctions, and increases vascular permeability, thereby facilitating the transmigration of leukocytes into lung tissue. At the molecular level, DNA damage and oxidative stress activate numerous signaling pathways, most notably the NF-κB signaling pathway, as well as pro-inflammatory mediators such as TGF-β, IL-1 and platelet-derived growth factor [159]. Persistent activation of these pathways promotes the transition from the inflammatory phase to fibrotic remodeling. ROS and inflammatory mediators can induce epithelial-to-mesenchymal transition (EMT) in alveolar epithelial cells and stimulate the proliferation of fibroblasts as well as their differentiation into myofibroblasts. Myofibroblasts produce large amounts of ECM components, including collagen and fibronectin, leading to excessive ECM deposition and lung tissue remodeling [160]. Simultaneously, TGF-β overexpression increases the levels of protease inhibitors, thereby limiting ECM degradation and promoting its accumulation [161]. Consequently, progressive fibrosis of the alveolar septa occurs, accompanied by thickening and stiffening of the lung parenchyma, reduction of alveolar spaces, and impaired gas exchange. Progressive remodeling of lung architecture ultimately leads to reduced respiratory capacity and loss of lung function [155].

Zhao et al. proposed the use of hydrogel as a radiation barrier in brachytherapy [162]. They engineered a composite hydrogel by self-assembling nickel nanoparticles on the surface of liquid metal particles and embedding them into an injectable hydrogel matrix. Results revealed that the hydrogel effectively protected surrounding healthy tissues from damage. At the same time, the embedded magnetic nanoparticles generated heat when exposed to a magnetic field, enabling controlled magnetic hyperthermia that can enhance the therapeutic efficacy against tumor cells.

In recent years, some research tested the use of hydrogels as materials supporting the protection and regeneration of lung tissue in the context of RILI. Zhou et al. demonstrated that lung tissue extracellular matrix-derived hydrogel can reduce the severity of lung damage after irradiation [163]. In the study, after irradiation rats received intratracheal injection of ECM-derived hydrogel. It was observed that hydrogel treatment improved the histopathological image of lung tissue and reduced pulmonary edema. The protective mechanism was associated with the inhibition of epithelial–mesenchymal transition and reduction in oxidative stress and levels of cytokines (TNF-α, IL-6, and TGF-β1). The gels also exerted an antifibrotic effect. Similar study was also performed to investigate the protective potential of a hydrogel composed of chitosan and tragacanth enriched with cellulose nanoparticles [164]. In the study performed on rat models, the hydrogel injection was performed intraperitoneally. The addition of cellulose nanoparticles increased the structural stability of the hydrogel. The formulation resulted in markedly lower levels of inflammation, mucus secretion, and hemorrhage within the lung tissue. In addition, the treatment alleviated the thickening of alveolar walls and reduced the radiation-induced increase in the thickness of the alveolar septum.

**Figure 6:**
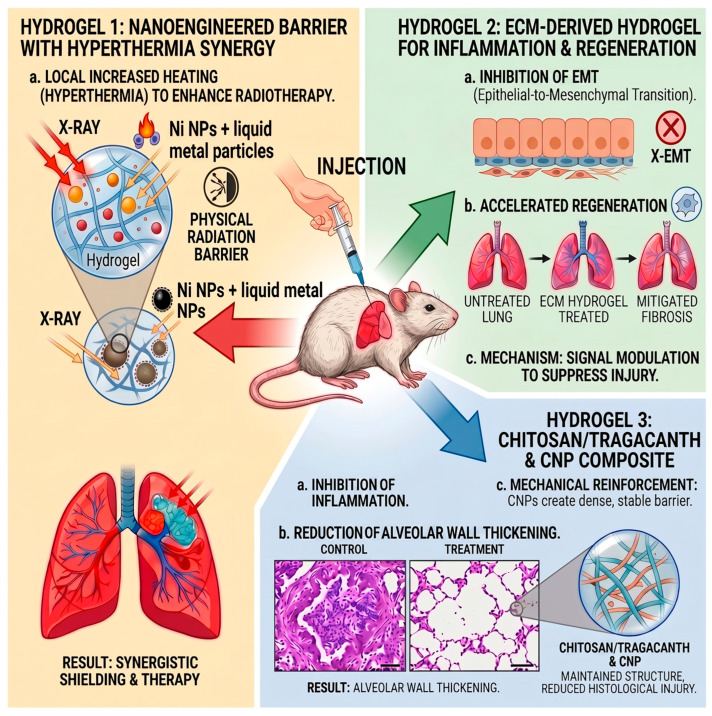
Multifunctional hydrogel strategies for radiation lung therapy. This platform utilizes nanoengineered barriers for radiotherapy shielding, ECM scaffolds for EMT inhibition and protection against fibrosis, and chitosan/CNP composites for reducing inflammation and alveolar thickening, enabling synergistic lung protection and repair transmission. The figure was created with the Artlist AI tool (AI Suite 16.5K), informed by the data and descriptions from the publications cited in the chapter.

The described hydrogel-based strategies for the prevention or treatment of RILI rely on injectable formulations (Figure 6). Such approaches are relatively invasive and may require specialized procedures, potentially limiting patient comfort and clinical feasibility, particularly when long-term treatment is required. Because of this, the development of a less invasive pulmonary delivery route is highly desirable. In the case of RILI the other way of hydrogel delivery can be inhalation. For example, microparticles from crosslinked hyaluronic acid have been proposed as carriers for controlled pulmonary drug delivery, demonstrating the feasibility of formulating hydrogel-derived particles suitable for aerosol administration and sustained drug release in lung tissue [165]. In addition, recent conceptual work has suggested that extracellular matrix-derived microgels, such as those based on amniotic ECM, could potentially be adapted for pulmonary delivery to mitigate RILI by modulating inflammatory responses and fibrotic remodeling processes [166].

### 4.5. Heart

**Figure 7:**
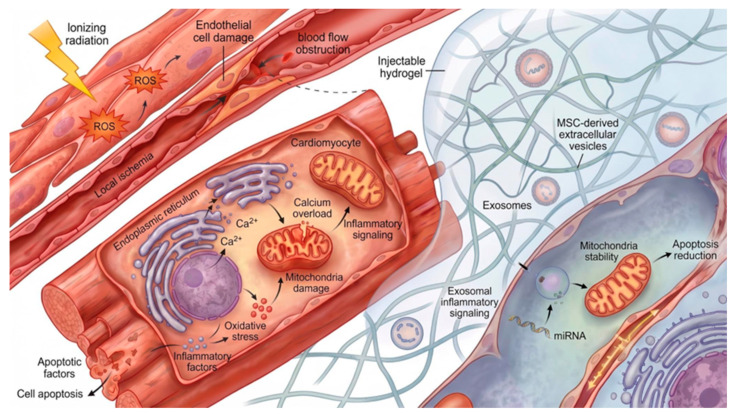
Radiation-induced cardiomyocyte damage and hydrogel-based therapeutic intervention. Radiation exposure leads to ROS generation, endothelial dysfunction, and microvascular ischemia. The figure was created with the Artlist AI tool (AI Suite 16.5K), informed by the data and descriptions from the publications cited in the chapter.

Radiation-induced heart disease (RIHD) is a complication following irradiation of chest tumors, such as Hodgkin’s lymphoma, esophageal cancer, lung cancer or breast cancer, and includes cardiac conditions such as cardiomyopathy, pericarditis, coronary artery disease, arrhythmia, valvular heart disease and conduction system abnormalities [167,168,169,170]. Endothelial cells in the capillaries of the myocardium are damaged by the absorption of ionizing radiation, which leads to the obstruction of microvascular circulation and, consequently, to ischemia and the development of myocardial fibrosis [171]. The mechanism of RIHD development is not fully understood. RIHD is likely the result of radiation-induced damage to cardiac cell organelles, including mitochondria and the endoplasmic reticulum. As cardiac myocytes receive repeated doses of radiation, the endoplasmic reticulum releases excessive amounts of calcium ions into the cytoplasm. This, in turn, leads to calcium overload in the mitochondria, which damages the cell membrane and releases factors leading to cell apoptosis. Chronic inflammation and excess ROS in the body, which are formed as a result of water breakdown under the influence of radiation, are also important for heart cells [167,170]. The degree of cell damage resulting from radiation exposure increases exponentially with increasing radiation doses [170]. Although modern radiotherapy methods administer lower doses of radiation to patients than in the past to reduce the risk of radiation complications as much as possible, this unfortunately does not eliminate the problem of RIHD occurrence [167,168]. Therefore, it is important to look for tools to combat the negative impact of radiation on heart cells when using radiotherapy. The key cellular and microvascular mechanisms underlying radiation-induced heart disease, together with emerging hydrogel-based therapeutic strategies, are illustrated in Figure 7.

Radiation-induced and ischemic cardiac injury are characterized by excessive production of reactive oxygen species (ROS), which aggravate cardiomyocyte apoptosis, inflammation, and adverse ventricular remodeling. Several injectable hydrogel systems have therefore been designed to attenuate oxidative stress and restore redox homeostasis within the injured myocardium. Fullerenol/alginate hydrogels employ fullerenol nanoparticles as potent ROS scavengers capable of neutralizing superoxide and hydroxyl radicals [172]. In addition to reducing oxidative stress, these hydrogels enhance the survival and cardiomyogenic differentiation of brown adipose-derived stem cells under hostile microenvironmental conditions. Similarly, melanin/alginate (MNPs/SA) hydrogels exploit the intrinsic antioxidant properties of cuttlefish-ink-derived melanin nanoparticles to eliminate excessive ROS accumulation [173]. Importantly, this system also exerts immunomodulatory effects by promoting macrophage polarization toward the reparative M2 phenotype, thereby coupling antioxidative activity with pro-healing immune regulation. The OGDPR hydrogel system, composed of oxidized xanthan gum, gelatin derivatives, and rosmarinic acid, was designed as a pH- and ROS-responsive conductive hydrogel capable of adapting to the evolving pathological microenvironment after myocardial injury [174]. The system enables controlled release of rosmarinic acid, a potent antioxidant and anti-inflammatory compound, while simultaneously supporting electrical integration and self-healing properties within damaged cardiac tissue. Melanin/polyvinyl alcohol (PVA) hydrogels combine the free-radical scavenging capacity of melanin nanoparticles with the mechanical stability of the PVA matrix [175]. Beyond antioxidative activity, melanin incorporation improves hydrogel conductivity, elasticity, and cell adhesion, thereby facilitating electromechanical integration and reducing infarct size and ventricular wall thinning following myocardial injury. Another multifunctional antioxidative platform is based on recombinant humanized type III collagen (rhCol III) combined with curcumin-loaded nanoparticles [176]. This myocardial infarction-responsive hydrogel integrates ECM-mimetic structural properties with controlled release of curcumin, resulting in antioxidative, anti-inflammatory, anti-apoptotic, and anti-fibrotic effects that collectively improve cardiac repair and remodeling outcomes.

Since persistent inflammation following radiation contributes to fibrosis, maladaptive remodeling, and progressive cardiac dysfunction, injectable hydrogels capable of modulating the inflammatory microenvironment have emerged as promising therapeutic platforms for myocardial repair. The ECM-NLC-colchicine system incorporates colchicine-loaded nanostructured lipid carriers into a decellularized extracellular matrix hydrogel to suppress inflammatory and fibrotic signaling pathways, including Wnt, Hippo, and TGF-β cascades [177]. This strategy enables early modulation of post-infarction immune remodeling while simultaneously attenuating fibrosis progression. Recombinant human collagen type I (rHCI) hydrogels have demonstrated strong immunomodulatory activity by increasing the abundance of pro-regenerative M2 macrophages within the ischemic region [178]. In parallel, these hydrogels reduce pathological monocyte recruitment, enhance capillary density, improve ventricular wall thickness, and limit adverse ventricular remodeling, thereby creating a more favorable reparative microenvironment. Oxidized/acylated hyaluronic acid (OHA/HHA) hydrogels were developed to protect mesenchymal stem cell aggregates from the hostile inflammatory milieu following myocardial infarction. In this system, poly(lactic-co-glycolic acid) (PLGA) microparticles modified with human VE-cadherin-Fc fusion protein (hVE-cad-Fc-PLGA) enhance cell–cell interactions and improve stem cell survival, while the hydrogel matrix itself provides temporary mechanical support and shields transplanted cells from inflammatory damage [179]. The Exo-PGN hydrogel system utilizes self-assembling peptide amphiphiles to deliver regenerative exosomes together with growth hormone-releasing peptide sequences (GHRPS) [180]. The incorporated GHRPS activates pro-survival signaling pathways while suppressing inflammation, fibrosis, and oxidative stress. Additionally, matrix metalloproteinase-2-responsive degradation sequences enable controlled hydrogel degradation and sustained exosome release within the injured myocardium.

Successful cardiac regeneration requires not only restoration of viable tissue but also recovery of the electrical conductivity and mechanical integrity of the myocardium. Consequently, many hydrogel systems have been engineered to simultaneously support cardiomyocyte survival, electrical coupling, angiogenesis, and structural reinforcement. To address impaired electrical signal propagation within fibrotic scar tissue, polypyrrole-chitosan (PPy:CHI) conductive hydrogels were developed to synchronize cardiomyocyte contractions and improve electrical coupling between adjacent cells [181]. These materials also enhance connexin-mediated gap junction communication, thereby supporting restoration of coordinated myocardial activity. PEG-PBA/HA-SH conductive hydrogels containing gold nanorods and astragaloside IV represent another multifunctional conductive platform [182]. Gold nanorods enhance electrical signal propagation and electromechanical integration, while astragaloside IV provides pro-angiogenic and anti-apoptotic activity. Together, these effects stimulate angiogenesis, improve intercellular signaling, and promote functional myocardial recovery. Similarly, oligo(polyethylene glycol fumarate) (OPF) hydrogels doped with graphene oxide (GO) nanoparticles provide both mechanical reinforcement and improved electrical conductivity [183]. GO incorporation facilitates electrical signal transmission within infarcted tissue and upregulates connexin-43 expression, thereby enhancing gap junction communication and improving cardiac function after myocardial infarction. Conductive GelMA/ODEX hydrogels form an interpenetrating polymer network characterized by enhanced energy dissipation, improved mechanical stability, and slower degradation kinetics [184]. These properties support the survival, proliferation, and cardiomyogenic differentiation of umbilical cord mesenchymal stem cells while simultaneously improving electrical integration within damaged myocardium. Temperature-sensitive chitosan/dextran/β-glycerophosphate (CS/DEX/β-GP) hydrogels function as injectable cell delivery systems that enhance stem cell retention and survival within damaged myocardium [185]. Dextran incorporation improves gelation kinetics and hydrogel stability, thereby strengthening the mechanical properties of the injectable scaffold and prolonging cell retention after transplantation. Fibrin hydrogels modified with vascular endothelial growth factor and platelet-derived growth factor enable localized and enzymatically controlled release of angiogenic factors through FXIIIa-sensitive covalent linkers [186]. This strategy promotes neovascularization, reduces fibrotic scar formation, and supports functional myocardial regeneration following injury. Matrigel-based scaffolds have also been employed to improve retention of adipose-derived stem cells, thereby enhancing neovascularization and preserving cardiac morphology and function after myocardial infarction [177]. However, despite their biological efficacy, Matrigel systems face significant translational limitations due to their tumor-derived origin, batch-to-batch variability, and potential risk of xenogenic contamination.

While earlier studies primarily focused on single-function or dual-function hydrogel systems, recent advances in biomaterials engineering have enabled the development of increasingly sophisticated multifunctional platforms integrating antioxidative, immunomodulatory, conductive, and regenerative properties. In 2025, Wang et al. developed an injectable sericin silk hydrogel OSA/SS-ADH/PPy@Exo, which could be a potential weapon in the fight against the effects of RIHD [168]. This hydrogel is composed of oxidized sodium alginate (OSA), a naturally occurring polymer characterized by high biocompatibility and water solubility, and sericin modified with adipic dihydrazide (SS-ADH). This last component has a positive effect on the mechanical properties of alginate hydrogel, improving its elasticity and flexibility, but also increasing the porosity of the hydrogel and its swelling capacity [187]. The gel also contains polypyrrole (PPy), which is a component that gives the hydrogel electrical conductivity. The key components of OSA/SS-ADH/PPy@Exo hydrogel are MSC exosomes extracted from the bone marrow stem cells. They can regulate the secretion of pro-inflammatory cytokines and reduce the level of ROS. Furthermore, they are characterized by high pro-regenerative abilities since miRNA contained in MSC exosomes promotes the repair of DNA fragments damaged by ionizing radiation. Notably, MSC-derived exosomes can cross biological barriers and exhibit low immunogenicity, allowing them to evade immune clearance and maintain a prolonged circulation half-life necessary to exert their therapeutic effects [168,188]. Owing to its pro-regenerative, antioxidant, and electroconductive properties, combined with a sustained release profile, the OSA/SS-ADH/PPy@Exo hydrogel represents a promising candidate for the treatment of RIHD.

### 4.6. Bones

The skeletal system is highly susceptible to radiation-induced complications [189,190]. Ionizing radiation triggers a cascade of detrimental effects, primarily mediated by persistent oxidative stress and vascular disruption. Elevated levels of ROS impair the proliferation and differentiation of bone marrow mesenchymal stem cells, which are essential for osteoblastogenesis [191]. This leads to a profound imbalance in bone remodeling: while osteoblast activity decreases, radiation simultaneously enhances osteoclast-mediated bone resorption [192,193]. The resulting deterioration of bone microarchitecture, characterized by reduced trabecular volume and increased spacing, significantly elevates the risk of osteoporosis and pathological fractures [191,194]. A critical factor in this pathology is vascular damage. Radiation-induced endothelial dysfunction leads to increased permeability, thrombosis, and impaired blood supply to bone tissue [195]. Reduced oxygen and nutrient delivery promotes tissue hypoxia and fibrosis, which can eventually culminate in osteoradionecrosis, a severe complication exemplified by radiation-associated injury of the mandible [196]. Although early clinical signs in the jaw may be limited, the rapid disruption of microvascular integrity and enhanced inflammatory signaling make therapeutic intervention in this area particularly challenging [192,193].

To address these challenges, recent research has focused on hydrogels that deliver therapeutic ions or proteins to the injured site. One of these ions is Mg^2+^. Magnesium is essential for normal bone physiology, yet its biological role within irradiated tissue remains incompletely understood [197]. Recent studies have reported that local administration of alginate-based hydrogels releasing Mg^2+^ can mitigate early bone loss, reduce apoptosis, and promote a reparative immune phenotype in models of radiation-induced bone injury [198]. The hydrogel was produced by crosslinking sodium alginate with magnesium ions (Mg@Alg), resulting in a stable three-dimensional polymeric network capable of controlled Mg^2+^ release. The mechanical behavior and degradation profile depended on the crosslinking degree, and the selected formulation enabled gradual release of most magnesium ions within two weeks, corresponding to the critical early phase of radiation response. Local administration increased magnesium levels in newly formed bone without affecting systemic mineral homeostasis, confirming good biocompatibility. In an animal model, Mg@Alg reduced post irradiation bone volume loss and improved trabecular microarchitecture [198]. Treated tissues exhibited fewer necrotic areas, decreased osteocyte apoptosis and attenuation of excessive osteoclast activation. Furthermore, increased microvessel density and improved vascular maturation were detected, which correlated with reduced DNA damage in well perfused regions.

Another reactive oxygen species-responsive hydrogel developed to enhance the efficacy of radiotherapy in the treatment of breast cancer bone metastases was a material referred to as R/P@Gel [199]. It was designed as a local drug delivery platform capable of releasing bioactive molecules directly within the tumor site. The hydrogel structure was based on chemically modified hyaluronic acid and polyvinyl alcohol, whose rapid gelation process allowed the system to be administered through injection and formed in situ at the target location. To confer therapeutic functionality, two bioactive molecules were incorporated into the hydrogel matrix: Toll-like receptor 7/8 agonist R848, which is capable of stimulating immune responses against tumor cells, and *S*-nitroso*-N*-acetyl-DL-penicillamine, a nitric oxide donor, which can promote vasodilation and improve tissue perfusion. In vivo experiments demonstrated that the combination of hydrogel administration and radiotherapy resulted in a more pronounced inhibition of tumor growth within bone compared with radiotherapy alone [199]. Histological analyses indicated improved microcirculation within the tumor area and enhanced tissue oxygenation, which may contribute to increased sensitivity of tumor cells to ionizing radiation. Furthermore, the application of the hydrogel caused increased infiltration of T lymphocytes and a reduction in immunosuppressive cell populations.

The composite hydrogel consisted of calcium alginate (CA) and amelogenin (AM). referred to as CA + AM hydrogel, combines structural support with biologically active components that promote bone regeneration and tissue healing [200]. Amelogenin, a protein found in developing enamel matrix, is known to stimulate the regeneration of periodontal tissues and bone [200]. The hydrogel was synthesized using hybrid and ionic cross-linking techniques, which allowed the formation of a stable three-dimensional structure. Scanning electron microscopy revealed a rough and porous surface morphology. This porous architecture is beneficial for tissue regeneration because it facilitates cell infiltration, nutrient diffusion, and oxygen exchange within the scaffold. The hydrogel demonstrated a high swelling capacity and appropriate mechanical strength. Degradation studies indicated that the hydrogel gradually decomposed, with about 60% of the hydrogel mass degraded during five weeks and the remaining portion degraded during a subsequent week. This degradation profile is advantageous for bone regeneration because it ensures mechanical stability of the hydrogel during the early stages of tissue formation and its gradual disappearing as new tissue develops. One of the most significant mechanisms underlying the activity of the CA + AM hydrogel involves the macrophage polarization toward the M2 phenotype [200]. This effect was associated with increased expression of anti-inflammatory markers such as interleukin-10, TGF-β1 and CD206. Additionally, the hydrogel directly stimulated osteogenesis by increasing the expression of osteogenic markers including Runx-2, ALP, OCN, and Col-1. The CA + AM hydrogel exhibited excellent biocompatibility and did not produce significant cytotoxic effects on cultured cells. In vitro studies revealed enhanced proliferation of mesenchymal stem cells and increased differentiation toward osteoblasts. Histological examination and micro-CT imaging confirmed significant bone formation in the hydrogel-treated groups [200].

**Figure 8:**
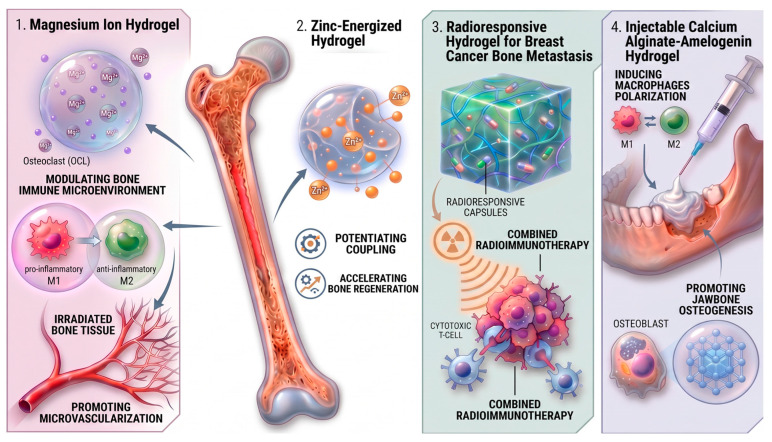
Schematic illustration of hydrogel-based strategies for bone regeneration. Zn-HG promotes angiogenesis–osteogenesis coupling, Mg-HG regulates immune–vascular responses, radiosensitive HA/PVA hydrogel supports tumor therapy, and injectable Ca-Alg/Am-HG enhances macrophage polarization and osteogenic differentiation. The figure was created with the Artlist AI tool (AI Suite 16.5K), informed by the data and descriptions from the publications cited in the chapter.

A zinc-energized dynamic composite hydrogel was designed to enhance the coupling between angiogenesis and osteogenesis during bone regeneration. The material consisted of a multi-component hydrogel system formed from gelatin methacryloyl (GelMA), oxidized hyaluronic acid and zinc-containing bioactive glass particles through dynamic Schiff-base reactions, creating a cross-linked hydrogel network with enhanced structural stability [201]. One important feature of the material was its sensitivity to acidic conditions. The microenvironment within bone defects is often characterized by reduced pH levels that are unfavorable for new bone formation. Under such conditions, the hydrogel structure gradually undergoes bond rearrangement, enabling the controlled release of zinc and silicon ions from the embedded bioactive glass particles. The biological activity of the developed hydrogel is based on the synergistic effects of several mechanisms. Zinc ions released from the material stimulate osteogenic differentiation of bone marrow mesenchymal stem cells and increase the activity of osteogenic markers such as alkaline phosphatase and bone matrix proteins. At the same time, zinc ions influence endothelial cells by promoting their migration and the formation of capillary-like structures. In vitro experiments demonstrated that the composite hydrogel exhibited favorable biocompatibility and supported cellular proliferation [201]. Cells cultured in the presence of the hydrogel showed increased alkaline phosphatase activity, formation of mineralized nodules, and enhanced expression of osteogenic genes, indicating a strong osteoinductive potential. Furthermore, the material significantly stimulated endothelial cell migration and tube formation, confirming its pro-angiogenic properties. The regenerative capacity of the hydrogel was further evaluated in vivo using a rat model of femoral bone defects [201]. Implantation of the hydrogel into the distal femoral defect resulted in enhanced formation of new bone tissue compared with control groups as well as increased vascularization within the defect region. Hydrogel-based strategies for bone regeneration are summarized in Figure 8.

Summarizing the hydrogel-based strategies developed for treating radiation-induced injuries across diverse tissues and organ systems, it is evident that these materials have evolved from simple protective barriers into multifunctional therapeutic platforms tailored to specific physiological requirements. By integrating bioactive agents, including antioxidants, stem cells, and growth factors, these systems actively modulate the local microenvironment to promote tissue repair and mitigate chronic complications. A comprehensive overview of these hydrogel-based treatments, categorized by tissue type along with their design strategies and key therapeutic outcomes, is presented in Table 3.

## 5. Challenges in Clinical Translation

While many advanced hydrogel systems are currently in preclinical development, their translational significance is underscored by a rapidly expanding clinical landscape. According to a recent comprehensive analysis by Dong et al. [202], there are currently over 80 active clinical trials investigating hydrogels for diverse therapeutic indications, ranging from oncology and ophthalmology to chronic wound care and musculoskeletal disorders. Furthermore, several hydrogel-based platforms have already achieved FDA approval as therapeutic carriers or medical devices, including products such as Seprafilm for adhesion prevention, Regranex for wound healing, and SpaceOAR for shielding healthy tissues during prostate cancer radiotherapy. These milestones demonstrate the successful integration of materials chemistry and biomedical engineering to meet stringent clinical and regulatory requirements.

However, the transition of hydrogel systems from preclinical models to routine clinical practice, particularly in fields such as radiotherapy, involves overcoming several significant hurdles. Successful translation hinges on four convergent parameters: biocompatibility, tunable degradation kinetics, mechanical compatibility, and scalable manufacturing. While laboratory results are promising, the following factors remain critical for successful commercialization and patient safety:

- Manufacturing scalability and reproducibility

Hydrogels, especially natural ones, often suffer from batch-to-batch variability [203]. The molecular weight, purity, and degree of substitution in polymers like hyaluronic acid or chitosan can vary depending on the source material. Scaling up production from benchtop quantities to industrial-scale batches under Good Manufacturing Practice conditions requires stringent quality control of rheological properties and gelation kinetics.

- Sterilization and structural integrity

Sterilization is a mandatory step that often compromises hydrogel stability. Standard autoclaving can cause thermal degradation, while gamma irradiation, though effective, induces chain scission or unwanted secondary cross-linking in natural polymers, altering the hydrogel’s mechanical shielding properties [204]. Alternative methods, such as ethylene oxide treatment or sterile filtration, pose risks of toxic residues or are limited only to low-viscosity precursors.

- Regulatory pathways

Regulatory divergence among international bodies like the FDA and EMA poses a formidable challenge for hydrogel commercialization. The absence of a unified protocol for safety assessment leads to a fragmented compliance environment, which inevitably prolongs the time-to-market and increases the overall cost of bringing these innovative materials into clinical practice.

- Long-term safety and biocompatibility

While rodent models provide initial safety data, they fail to replicate the human long-term response to degradation by-products. In radiotherapy, the hydrogel must maintain its position for the duration of the treatment (usually 5–8 weeks) and then biodegrade without triggering chronic inflammation or fibrous encapsulation, which could interfere with subsequent imaging or late-tissue monitoring.

Overcoming these translational barriers is essential to transform the advanced hydrogel biomaterials into accessible clinical tools that can significantly improve outcomes for patients undergoing radiotherapy.

## 6. Conclusions and Future Perspectives

The therapeutic management of radiation-induced wounds represents a unique challenge due to the complex interplay of chronic oxidative stress, impaired vascularization, and a persistent inflammatory microenvironment. Traditional wound gauze dressings often fail in these scenarios, as they cannot address the deep-seated cellular dysfunction characteristic of irradiated tissues. Furthermore, they require frequent replacement to prevent maceration and adhesion to the wound surface, which often leads to painful dressing changes and mechanical trauma to the fragile, irradiated epithelium. The recent development of bioactive and responsive hydrogels has opened new avenues for effective intervention. Modern hydrogel materials are no longer merely passive physical barriers but instead, they address the fundamental requirements that traditional dressings neglect. As evidenced by recent studies, hydrogels provide essential wettability, facilitate gas exchange, and enhance autolytic debridement [205]. Their primary advantage, however, lies in their versatility as targeted delivery systems for antibiotics and nanoparticles to manage the high risk of infection in immunocompromised irradiated tissues, as well as growth factors and regulatory peptides that trigger regeneration in dormant irradiated cells. Moreover, the hydrogels serve as dynamic scaffolds that can actively neutralize ROS, restore microcirculation, and re-establish the balance between tissue formation and destruction. Nevertheless, the transition from traditional gauze to advanced hydrogels is not without limitations. While hydrogels offer superior biomechanical properties and painlessness, their clinical application in radiotherapy is often hindered by higher production costs and the need for specialized sterilization methods that do not compromise the integrity of encapsulated bioactives [206]. Despite these challenges, the shift toward bioactive and responsive hydrogels represents a necessary evolution. Looking forward, the next generation of hydrogel systems must evolve beyond single-function materials toward intelligent, adaptive platforms. This includes the integration of real-time biosensors capable of monitoring the wound microenvironment (e.g., pH, temperature, or specific biomarkers) to trigger on-demand drug release. Furthermore, the application of 3D bioprinting and artificial intelligence in scaffold design offers the potential for patient-specific, “tailor-made” hydrogels that match the precise geometry and severity of the radiation-induced defect. However, realizing this potential will require a concerted effort to standardize large-animal clinical trials and harmonize global regulatory frameworks to ensure that these complex technologies can be manufactured consistently and affordably. Addressing these translational gaps will be pivotal in moving from experimental successes to routine clinical implementation. Future research should prioritize the development of multi-functional, injectable systems that can be easily applied in oncological settings, ensuring that cancer survivors achieve a significantly better quality of life through enhanced tissue repair.

## Figures and Tables

**Table 1:** Comprehensive overview of the advantages and limitations of hydrogels in radiation-induced wound management.

Feature/Property	Mechanistic Basis	Clinical/Functional Advantage	Relevance in Radiation Injury	Limitations/Challenges	Refs.
High water content (>90%)	Hydrophilic polymer network retaining large volumes of water	Maintains moist wound environment, reduces pain, promotes autolytic debridement	Prevents desiccation of irradiated tissues and supports re-epithelialization	Limited absorption in highly exudative wounds; risk of maceration	[5,7,8]
Extracellular matrix (ECM) mimicry	3D porous structure resembling native ECM	Supports cell adhesion, migration, and proliferation	Critical for regeneration of radiation-damaged tissues with impaired ECM	May lack sufficient mechanical strength for load-bearing sites	[2,9]
Tunable mechanical properties	Adjustable crosslink density and polymer composition	Customizable stiffness and elasticity for different tissues	Enables adaptation to fragile, radiation-damaged tissues	Overly soft gels may degrade rapidly or lack durability	[1,10]
Biocompatibility	Use of natural/synthetic non-toxic polymers	Minimal immune response, safe for long-term application	Essential for repeated use in radiotherapy patients	Natural materials may vary batch-to-batch	[7,11]
Stimuli-responsiveness	Sensitivity to pH, ROS, temperature, or enzymes	Controlled and targeted drug release	Allows site-specific therapy in ROS-rich radiation environment	Complex synthesis and potential instability	[6,12]
Drug delivery capability	Encapsulation and sustained release of bioactive agents	Localized delivery of antioxidants, growth factors, antibiotics	Reduces systemic toxicity and enhances therapeutic efficiency	Drug release kinetics may be difficult to control precisely	[11,13]
ROS-scavenging capacity	Incorporation of antioxidants or catalytic nanoparticles	Neutralizes oxidative stress at injury site	Directly addresses primary mechanism of radiation damage	Limited duration of activity; depletion over time	[13,14,15]
Immunomodulatory effects	Regulation of cytokine release and macrophage polarization	Reduces chronic inflammation and fibrosis	Crucial in preventing non-healing radiation wounds	Requires precise temporal control of immune response	[16,17,18]
Non-adhesive/ atraumatic removal	Soft, hydrated surface	Reduces pain during dressing changes	Important for sensitive irradiated skin	May require fixation in mobile anatomical areas	[7]
Cooling and soothing effect	Water evaporation and thermal buffering	Immediate pain relief and patient comfort	Beneficial in acute radiation dermatitis	Temporary effect only	[7]
Oxygen and nutrient permeability	Porous structure	Supports cellular metabolism and healing	Important in hypoxic radiation-damaged tissues	Limited in thicker or highly crosslinked systems	[5]
Cost and scalability	Advanced synthesis and functionalization			Higher cost than conventional dressings	[10]

**Table 2:** Mechanism-based classification of hydrogels used in radiation-induced tissue repair.

Hydrogel Category	Key Mechanism of Action	Therapeutic Targets	Key Advantages	Limitations	Refs.
ROS-scavenging hydrogels	Neutralization of ROS, interruption of oxidative stress cascade	DNA damage, lipid peroxidation, chronic inflammation	Modulate oxidative microenvironment; prevent chronic injury progression	Limited duration of antioxidant effect; potential degradation under radiation	[13,14,15]
Immuno-modulatory hydrogels	Regulation of macrophage polarization (M1 → M2), cytokine modulation	Chronic inflammation, fibrosis, impaired healing	Restore immune balance; promote angiogenesis and ECM remodeling	Complex design; temporal control required for optimal efficacy	[16,26,27,28,29,30,31]
Proangiogenic hydrogels	Controlled delivery of VEGF and angiogenic factors; endothelial activation	Ischemia, vascular damage, impaired perfusion	Enhance neovascularization and tissue oxygenation	Risk of uncontrolled angiogenesis; stability issues	[32,33,34,35,36]
Regenerative/ECM-mimicking hydrogels	Structural mimicry of ECM; promotion of cell adhesion and migration	Tissue remodeling, fibrosis, impaired regeneration	Support full tissue reconstruction; integrate multiple repair pathways	Manufacturing complexity; variability of natural materials	[37,38,39]

**Table 3:** Hydrogel-based treatments for radiation-induced injuries classified by tissue/organs.

Tissue/Organ	Hydrogel Design Strategy	Examples of Hydrogel Systems	Key Therapeutic Outcomes	Key Challenges	Refs.
Skin	Antioxidant, regenerative, multifunctional hydrogels	Carbomer-FA, IFI6-PDA/alginate, PHF@Res, ADM hydrogels, Janus polypeptide	Accelerated wound closure, increased collagen deposition, reduced inflammation	Mechanical durability, stability under radiation	[13,14,15,54,90,95]
Mucosa (oral, esophageal)	Mucoadhesive, antimicrobial, ROS-responsive hydrogels	QTMP-Gel (quaternized chitosan-tannic acid), PFP-BA@Gel, MSC-loaded HA/silanized HPMC, oCP@As	Prolonged retention, reduced ulceration, improved healing and infection control	Dynamic environment (saliva, movement), drug washout	[12,100,103,104,105,107,108,109]
Gastrointestinal tract	Spacer hydrogels, cell-delivery scaffolds, drug-delivery systems	SpaceOAR (PEG spacer), Si-HPMC + MSCs, silk-elastin-like protein + glycosaminoglycans, PEG-4MAL + organoids	Reduced radiation dose to healthy tissue, improved epithelial repair, reduced toxicity	Invasive placement (spacers), material displacement	[107,126,127,144,145,149]
Lungs	Injectable ECM-mimicking and anti-fibrotic hydrogels	Lung ECM-derived hydrogel, Chitosan-tragacanth + cellulose NPs	Reduced fibrosis, improved lung structure, decreased cytokine levels	Invasive delivery, limited clinical translation	[163,164]
Heart	Injectable conductive and exosome-loaded hydrogels	OSA/SS-ADH/PPy@Exo (sericin silk + MSC exosomes)	Reduced ROS, improved cardiac repair, enhanced electrical conductivity	Limited data, early-stage research	[168]
Bone	Ion-releasing, osteoinductive, immunomodulatory hydrogels	Mg@Alg, CA + AM (calcium alginate + amelogenin), R/P@Gel (HA-based ROS-responsive), Zn-energized GelMA/HA-CHO	Enhanced osteogenesis, improved vascularization, reduced bone loss	Complex microenvironment, long healing time	[198,199,201]

## Data Availability

No new data were created or analyzed in this study.

## Abbreviations

The following abbreviations are used in this manuscript:

ADSC	Adipose-derived stem cell
eNOS	Endothelial nitric oxide synthase
ECM	Extracellular matrix
EMT	Epithelial-to-mesenchymal transition
GF	Growth factor
IL	Interleukin
JAK/STAT	Janus kinase/signal transducer and activator of transcription
MSC	Mesenchymal stem cell
NF-kB	Nuclear factor kappa-light-chain-enhancer of activated B cells
PEG	Polyethylene glycol
RIHD	Radiation-induced heart disease
RILI	Radiation-induced lung injury
RIOM	Radiation-induced oral mucositis
ROS	Reactive oxygen species
TGF	Transforming growth factor
TNF	Tumor necrosis factor
VEGF	Vascular endothelial growth factor

## References

[1] Ho T.C., Chang C.C., Chan H.P., Chung T.W., Shu C.W., Chuang K.P., Duh T.H., Yang M.H., Tyan Y.C. (2022). Hydrogels: Properties and Applications in Biomedicine. Molecules.

[2] Ahmed E.M. (2015). Hydrogel: Preparation, characterization, and applications: A review. J. Adv. Res..

[3] Wichterle O., Lím D. (1960). Hydrophilic Gels for Biological Use. Nature.

[4] Segneanu A.E., Bejenaru L.E., Bejenaru C., Blendea A., Mogoşanu G.D., Biţă A., Boia E.R. (2025). Advancements in Hydrogels: A Comprehensive Review of Natural and Synthetic Innovations for Biomedical Applications. Polymers.

[5] Xiang J., Shen L., Hong Y. (2020). Status and future scope of hydrogels in wound healing: Synthesis, materials and evaluation. Eur. Polym. J..

[6] Cao H., Duan L., Zhang Y., Cao J., Zhang K. (2021). Current hydrogel advances in physicochemical and biological response-driven biomedical application diversity. Signal Transduct. Target. Ther..

[7] Song A., Rane A.A., Christman K.L. (2012). Antibacterial and cell-adhesive polypeptide and poly(ethylene glycol) hydrogel as a potential scaffold for wound healing. Acta Biomater..

[8] Gounden V., Singh M. (2024). Hydrogels and Wound Healing: Current and Future Prospects. Gels.

[9] Mottareale R., Frascogna C., La Verde G., Arrichiello C., Muto P., Netti P.A., Fusco S., Panzetta V., Pugliese M. (2024). Impact of ionizing radiation on cell-ECM mechanical crosstalk in breast cancer. Front. Bioeng. Biotechnol..

[10] Britto E.J., Nezwek T.A., Popowicz P., Robins M. (2025). Wound Dressings 2024. StatPearls.

[11] Su Y., Cui H., Yang C., Li L., Xu F., Gao J., Zhang W. (2022). Hydrogels for the treatment of radiation-induced skin and mucosa damages: An up-to-date overview. Front. Mater..

[12] Shi M., Huang C., Peng Y., Yang C., Sun M. (2026). Multifunctional Hydrogel with Dual Functions of ROS Scavenging and Responsive Antibiotic Release for Synergistic Oral Mucositis Treatment. ACS Omega.

[13] Shen J., Jiao W., Yang J., Zhuang B., Du S., Wu Y., Huang G., Zhang Y., Wang Y., Xu C. (2025). In situ photocrosslinkable hydrogel treats radiation-induced skin injury by ROS elimination and inflammation regulation. Biomaterials.

[14] Huang C., Huangfu C., Bai Z., Zhu L., Shen P., Wang N., Li G., Deng H., Ma Z., Zhou W. (2024). Multifunctional carbomer based ferulic acid hydrogel promotes wound healing in radiation-induced skin injury by inactivating NLRP3 inflammasome. J. Nanobiotech..

[15] Hao J., Sun M., Li D., Zhang T., Li J., Zhou D. (2022). An IFI6-based hydrogel promotes the healing of radiation-induced skin injury through regulation of the HSF1 activity. J. Nanobiotech..

[16] Mai Y., Wang H., Lu J., Shi S., Cai Y., Zhang W., Xie S., Huang R., Ji S., Qu X. (2025). Catalyst-modulated hydrogel dynamics for decoupling viscoelasticity and directing macrophage fate for diabetic wound healing. Bioact. Mater..

[17] Chou R., Dana T., Bougatsos C., Blazina I., Starmer A.J., Reitel K., Buckley D.I. (2013). Pressure ulcer risk assessment and prevention: A systematic comparative effectiveness review. Ann. Intern. Med..

[18] Tian R., Liu J., Dou G., Lin B., Chen J., Yang G., Li P., Liu S., Jin Y., Qiu X. (2022). Synergistic antibiosis with spatiotemporal controllability based on multiple-responsive hydrogel for infectious cutaneous wound healing. Smart Mater. Med..

[19] Macmillan M.S., Wells M., MacBride S., Raab G.M., Munro A., MacDougall H. (2007). Randomized comparison of dry dressings versus hydrogel in management of radiation-induced moist desquamation. Int. J. Radiat. Oncol. Biol. Phys..

[20] Demeter M., Scărișoreanu A., Călina I. (2023). State of the Art of Hydrogel Wound Dressings Developed by Ionizing Radiation. Gels.

[21] Nagasawa N., Mitomo H., Yoshii F., Kume T. (2000). Radiation-induced degradation of sodium alginate. Polym. Degrad. Stab..

[22] Srinivas A., Ramamurthi A. (2007). Effects of gamma-irradiation on physical and biologic properties of crosslinked hyaluronan tissue engineering scaffolds. Tissue Eng..

[23] Wang Y., Liu H., He Y., Li M., Gao J., Han Z., Zhou J., Li J. (2025). Hydrogel-Based Strategies for the Prevention and Treatment of Radiation-Induced Skin Injury: Progress and Mechanistic Insights. Biomimetics.

[24] Ahmad Z., Salman S., Khan S.A., Amin A., Rahman Z.U., Al-Ghamdi Y.O., Akhtar K., Bakhsh E.M., Khan S.B. (2022). Versatility of Hydrogels: From Synthetic Strategies, Classification, and Properties to Biomedical Applications. Gels.

[25] Ullah F., Othman M.B., Javed F., Ahmad Z., Md Akil H. (2015). Classification, processing and application of hydrogels: A review. Mater. Sci. Eng. C Mater. Biol. Appl..

[26] Huang R., Sun W., Li W., Hu R., Meng R., Peng Z., Yang R., Huang T., Du J., Shang L. (2024). Immunomodulatory hydrogel patches loaded with curcumin and tannic acid assembled nanoparticles for radiation dermatitis repair and radioprotection. Chem. Eng. J..

[27] Fu Y.J., Shi Y.F., Wang L.Y., Zhao Y.F., Wang R.K., Li K., Zhang S.T., Zha X.J., Wang W., Zhao X. (2023). All-Natural Immunomodulatory Bioadhesive Hydrogel Promotes Angiogenesis and Diabetic Wound Healing by Regulating Macrophage Heterogeneity. Adv. Sci..

[28] Jin C., Liang J., Wu J., Han X., Zhou Y., Li B., Sun W., Su J., Sun J., Wan S. (2026). Temporal Immunomodulatory Hydrogel Regulating the Immune-Osteogenic Cascade for Infected Bone Defects Regeneration. Adv. Mater..

[29] Yang P., Hu Y., Ju Y., Hsiung N., Ye J., Jian A., Lei L., Fang B. (2026). DNA-Inspired Multi-Functional Double-Cross-Linking Self-Healing Hydrogel Promotes the Healing of Diabetic Wounds. Adv. Sci..

[30] Mehnath S., Karthikeyan K., Jeyaraj M. (2024). Mechanical Force on Hydrogel Implication on Enhanced Drug Release, Antibacterial, and M2 Macrophage Polarization: New Insights Alleviate Diabetic Wound Healing. ACS Appl. Mater. Interfaces.

[31] Zhou P., Yan B., Wei B., Fu L., Wang Y., Wang W., Zhang L., Mao Y. (2023). Quercetin-solid lipid nanoparticle-embedded hyaluronic acid functionalized hydrogel for immunomodulation to promote bone reconstruction. Regen. Biomater..

[32] Zhang W., Qian S., Chen J., Jian T., Wang X., Zhu X., Dong Y., Fan G. (2024). Photo-Crosslinked Pro-Angiogenic Hydrogel Dressing for Wound Healing. Int. J. Mol. Sci..

[33] Hu Y., Wu B., Xiong Y., Tao R., Panayi A.C., Chen L., Tian W., Xue H., Shi L., Zhang X. (2021). Cryogenic 3D printed hydrogel scaffolds loading exosomes accelerate diabetic wound healing. Chem. Eng. J..

[34] Siebert L., Luna-Cerón E., García-Rivera L.E., Oh J., Jang J., Rosas-Gómez D.A., Pérez-Gómez M.D., Maschkowitz G., Fickenscher H., Oceguera-Cuevas D. (2021). Light-controlled growth factors release on tetrapodal ZnO-incorporated 3D-printed hydrogels for developing smart wound scaffold. Adv. Funct. Mater..

[35] Tao B., Lin C., Qin X., Yu Y., Guo A., Li K., Tian H., Yi W., Lei D., Chen Y. (2022). Fabrication of gelatin-based and Zn2+-incorporated composite hydrogel for accelerated infected wound healing. Mater. Today Bio.

[36] Li W., Xie H., Gou L., Zhou Y., Wang H., Li R., Zhang Y., Liu S., Liu J., Lu Y. (2023). DNA-Based Hydrogels with Multidrug Sequential Release for Promoting Diabetic Wound Regeneration. JACS Au.

[37] Wang W., Cheng Z., Yu M., Liu K., Duan H., Zhang Y., Huang X., Li M., Li C., Hu Y. (2025). Injectable ECM-mimetic dynamic hydrogels abolish ferroptosis-induced post-discectomy herniation through delivering nucleus pulposus progenitor cell-derived exosomes. Nat. Commun..

[38] Jin W., Li Y., Yu M., Ren D., Han C., Guo S. (2025). Advances of exosomes in diabetic wound healing. Burns Trauma.

[39] Hao Y., Li H., Guo J., Wang D., Zhang J., Liu J., Yang C., Zhang Y., Li G., Liu J. (2023). Bio-Inspired Antioxidant Heparin-Mimetic Peptide Hydrogel for Radiation-Induced Skin Injury Repair. Adv. Health Mater..

[40] Xue J., Yu C., Tang Y., Mo W., Tang Z., Sheng W., Jiao Y., Zhu W., Cao J. (2021). NF-E2-Related Factor 2 (Nrf2) Ameliorates Radiation-Induced Skin Injury. Front. Oncol..

[41] Averbeck D., Rodriguez-Lafrasse C. (2021). Role of Mitochondria in Radiation Responses: Epigenetic, Metabolic, and Signaling Impacts. Int. J. Mol. Sci..

[42] Azzam E.I., Jay-Gerin J.P., Pain D. (2012). Ionizing radiation-induced metabolic oxidative stress and prolonged cell injury. Cancer Lett..

[43] Liu C., Wei J., Wang X., Zhao Q., Lv J., Tan Z., Xin Y., Jiang X. (2024). Radiation-induced skin reactions: Oxidative damage mechanism and antioxidant protection. Front. Cell Dev. Biol..

[44] Tang R., Yin J., Liu Y., Xue J. (2024). FLASH radiotherapy: A new milestone in the field of cancer radiotherapy. Cancer Lett..

[45] Calaf G.M., Crispin L.A., Muñoz J.P., Aguayo F., Narayan G., Roy D. (2022). Cell Adhesion Molecules Affected by Ionizing Radiation and Estrogen in an Experimental Breast Cancer Model. Int. J. Mol. Sci..

[46] Melia E., Parsons J.L. (2023). DNA damage and repair dependencies of ionising radiation modalities. Biosci. Rep..

[47] Dent P., Yacoub A., Fisher P.B., Hagan M.P., Grant S. (2003). MAPK pathways in radiation responses. Oncogene.

[48] Liermann-Wooldrik K.T., Kosmacek E.A., McDowell J.A., Takkar S., Murthy D., Singh P.K., Schott M.B., Ponnusamy M.P., Oberley-Deegan R.E. (2025). Radiation Promotes Acute and Chronic Damage to Adipose Tissue. Int. J. Mol. Sci..

[49] Wei J., Wang B., Wang H., Meng L., Zhao Q., Li X., Xin Y., Jiang X. (2019). Radiation-Induced Normal Tissue Damage: Oxidative Stress and Epigenetic Mechanisms. Oxid. Med. Cell Longev..

[50] Cheng J., Dong J., Fang Y., Zhang X., Dang X. (2026). Radiation-induced skin injury: A review of pathophysiology, assessment, management, and re-irradiation protocols. Front. Oncol..

[51] Cui J., Wang T.J., Zhang Y.X., She L.Z., Zhao Y.C. (2024). Molecular biological mechanisms of radiotherapy-induced skin injury occurrence and treatment. Biomed. Pharmacother..

[52] Zhou L., Zhu J., Liu Y., Zhou P.K., Gu Y. (2024). Mechanisms of radiation-induced tissue damage and response. MedComm.

[53] Martin M.T., Vulin A., Hendry J.H. (2016). Human epidermal stem cells: Role in adverse skin reactions and carcinogenesis from radiation. Mutat. Res. Rev. Mutat. Res..

[54] Liu Z., Gu J., Gao Y., Hu H., Jiang H. (2025). ADSC-derived exosomes mitigate radiation-induced skin injury by reducing oxidative stress, inflammation and cell death. Front. Public Health.

[55] Nathan C., Ding A. (2010). Nonresolving inflammation. Cell.

[56] Peña O.A., Martin P. (2024). Cellular and molecular mechanisms of skin wound healing. Nat. Rev. Mol. Cell Biol..

[57] Murray P.J., Allen J.E., Biswas S.K., Fisher E.A., Gilroy D.W., Goerdt S., Gordon S., Hamilton J.A., Ivashkiv L.B., Lawrence T. (2014). Macrophage activation and polarization: Nomenclature and experimental guidelines. Immunity.

[58] Au N.P.B., Wu T., Kumar G., Jin Y., Li Y.Y.T., Chan S.L., Lai J.H.C., Chan K.W.Y., Yu K.N., Wang X. (2024). Low-dose ionizing radiation promotes motor recovery and brain rewiring by resolving inflammatory response after brain injury and stroke. Brain Behav. Immun..

[59] Chen J., Liu X., Zeng Z., Li J., Luo Y., Sun W., Gong Y., Zhang J., Wu Q., Xie C. (2020). Immunomodulation of NK Cells by Ionizing Radiation. Front. Oncol..

[60] Nakashima H., Nakashima M., Kinoshita M., Ikarashi M., Miyazaki H., Hanaka H., Imaki J., Seki S. (2016). Activation and increase of radio-sensitive CD11b+ recruited Kupffer cells/macrophages in diet-induced steatohepatitis in FGF5 deficient mice. Sci. Rep..

[61] Gupta K., Vuckovic I., Zhang S., Xiong Y., Carlson B.L., Jacobs J., Olson I., Petterson X.M., Macura S.I., Sarkaria J. (2020). Radiation Induced Metabolic Alterations Associate with Tumor Aggressiveness and Poor Outcome in Glioblastoma. Front. Oncol..

[62] Yi M., Li T., Niu M., Zhang H., Wu Y., Wu K., Dai Z. (2024). Targeting cytokine and chemokine signaling pathways for cancer therapy. Signal Transduct. Target. Ther..

[63] Abe J.I., Allen B.G., Beyer A.M., Lewandowski D., Mapuskar K.A., Subramanian V., Tamplin M.R., Grumbach I.M. (2024). Radiation-Induced Macrovessel/Microvessel Disease. Arterioscler. Thromb. Vasc. Biol..

[64] Choi D.H., Oh D., Na K., Kim H., Choi D., Jung Y.H., Ahn J., Kim J., Kim C.H., Chung S. (2023). Radiation induces acute and subacute vascular regression in a three-dimensional microvasculature model. Front. Oncol..

[65] Lee M.O., Song S.H., Jung S., Hur S., Asahara T., Kim H., Kwon S.M., Cha H.J. (2012). Effect of ionizing radiation induced damage of endothelial progenitor cells in vascular regeneration. Arterioscler. Thromb. Vasc. Biol..

[66] Lee W.H., Cho H.J., Sonntag W.E., Lee Y.W. (2011). Radiation attenuates physiological angiogenesis by differential expression of VEGF, Ang-1, tie-2 and Ang-2 in rat brain. Radiat. Res..

[67] Aktaş C., Kurtman C., Ozbilgin M.K., Tek I., Toprak S.K. (2013). An Experimental Study of Radiation Effect on Normal Tissue: Analysis of HIF-1α, VEGF, eIF2, TIA-1, and TSP-1 Expression. Turk. J. Haematol..

[68] Müller-Seubert W., Ostermaier P., Horch R.E., Distel L., Frey B., Erber R., Arkudas A. (2023). The Influence of Different Irradiation Regimens on Inflammation and Vascularization in a Random-Pattern Flap Model. J. Pers. Med..

[69] Ria R., Cirulli T., Giannini T., Bambace S., Serio G., Portaluri M., Ribatti D., Vacca A., Dammacco F. (2008). Serum levels of angiogenic cytokines decrease after radiotherapy in non-Hodgkin lymphomas. Clin. Exp. Med..

[70] Heissig B., Rafii S., Akiyama H., Ohki Y., Sato Y., Rafael T., Zhu Z., Hicklin D.J., Okumura K., Ogawa H. (2005). Low-dose irradiation promotes tissue revascularization through VEGF release from mast cells and MMP-9-mediated progenitor cell mobilization. J. Exp. Med..

[71] Veith A.P., Henderson K., Spencer A., Sligar A.D., Baker A.B. (2019). Therapeutic strategies for enhancing angiogenesis in wound healing. Adv. Drug Deliv. Rev..

[72] Lee C., Shim S., Jang H., Myung H., Lee J., Bae C.H., Myung J.K., Kim M.J., Lee S.B., Jang W.S. (2017). Human umbilical cord blood-derived mesenchymal stromal cells and small intestinal submucosa hydrogel composite promotes combined radiation-wound healing of mice. Cytotherapy.

[73] Zhu Y., Ma Z., Kong L., He Y., Chan H.F., Li H. (2020). Modulation of macrophages by bioactive glass/sodium alginate hydrogel is crucial in skin regeneration enhancement. Biomaterials.

[74] Nie S., Ren C., Liang X., Cai H., Sun H., Liu F., Ji K., Wang Y., Liu Q. (2022). Supramolecular Hydrogel-Wrapped Gingival Mesenchymal Stem Cells in Cutaneous Radiation Injury. Cell.

[75] Cytlak U.M., Dyer D.P., Honeychurch J., Williams K.J., Travis M.A., Illidge T.M. (2022). Immunomodulation by radiotherapy in tumour control and normal tissue toxicity. Nat. Rev. Immunol..

[76] Salvo N., Barnes E., van Draanen J., Stacey E., Mitera G., Breen D., Giotis A., Czarnota G., Pang J., De Angelis C. (2010). Prophylaxis and management of acute radiation-induced skin reactions: A systematic review of the literature. Curr. Oncol..

[77] Cui X., Wang J., Xu X., Cao X.P., Zhou Y., Guo J. (2025). Progress and Application of Multifunctional Hydrogel in Radioactive Skin Injury. Adv. Mater. Interfaces.

[78] Han X., Zhou C., Xu R., Jia Z., Liu Y., Chen S., Tang W., Li X., Zhou L., Sun Y. (2025). Functionalized hydrogel sequentially deliver tannic acid and bioactive probiotics for radiation-induced skin injury. Mater. Today Bio.

[79] Zhu M., Shi G., Chen R., Li Z., Wang J., Wu Z., Guo L., Wei Y., Li J. (2025). A multifunctional parathyroid hormone-related supramolecular peptide-loaded dual network hydrogel for radiation-induced wound repair. Mater. Today Bio.

[80] Zhu W., Jia L., Chen G., Zhao H., Sun X., Meng X., Zhao X., Xing L., Yu J., Zheng M. (2016). Epigallocatechin-3-gallate ameliorates radiation-induced acute skin damage in breast cancer patients undergoing adjuvant radiotherapy. Oncotarget.

[81] Nam S., Smith D.M., Dou Q.P. (2001). Ester bond-containing tea polyphenols potently inhibit proteasome activity in vitro and in vivo. J. Biol. Chem..

[82] Wang J., Gao L., Song J., Li S. (2023). Study of EGCG composite hydrogel for the treatment of radiation-induced skin injuries. J. Appl. Biomater. Funct. Mater..

[83] Dong L., Jia R., Liu Z., Aiyiti W., Shuai C., Li Z., Fu Q., Li X. (2024). Tannic acid based multifunctional hydrogels with mechanical stability for wound healing. Colloids Surf. B Biointerfaces.

[84] Fang D., Chen S., Wu C., Zuo J., Wang W., Zhang Y., Liu J., Feng H., Chu W., Jin Y. (2026). In situ photocrosslinking ROS-adaptive caffeoyl chitosan/boronic acid-grafted gelatin hydrogels for treatment of combined radiation-burn injury. Biomater. Adv..

[85] Wen Y., Wang Y., Zhao C., Zhao B., Wang J. (2023). The Pharmacological Efficacy of Baicalin in Inflammatory Diseases. Int. J. Mol. Sci..

[86] Liu X., Shu W., Zhong Q., Zeng A., Zeng Y., Gu H., Chen P., Li X. (2025). A Baicalin Liposome-Based Temperature-Sensitive Hydrogel for Treating Ultraviolet-Induced Skin Damage. Int. J. Nanomed..

[87] Dalcin A.J.F., Roggia I., Felin S., Vizzotto B.S., Mitjans M., Vinardell M.P., Schuch A.P., Ourique A.F., Gomes P. (2021). UVB photoprotective capacity of hydrogels containing dihydromyricetin nanocapsules to UV-induced DNA damage. Colloids Surf. B Biointerfaces.

[88] de Araújo Andrade T., Heimfarth L., Dos Santos D.M., Dos Santos M.R.V., de Albuquerque-Júnior R.L.C., Dos Santos-Neto A.G., de Araujo G.R.S., Lira A.A.M., Matos S.S., Frank L.A. (2022). Hesperetin-Based Hydrogels Protect the Skin against UV Radiation-Induced Damage. AAPS PharmSciTech.

[89] Dong J., Lang Y., He J., Cui J., Liu X., Yuan H., Li L., Zhou M., Wang S. (2025). Phycocyanin-based multifunctional microspheres for treatment of infected radiation-induced skin injury. Biomaterials.

[90] Liu X., Guo T., Huang Z., Chen S., Chen L., Li C., Tian T., Qian Y., Yang L., Xiang J. (2024). Acellular dermal matrix hydrogels promote healing of radiation-induced skin injury in a rat model. J. Mater. Chem. B.

[91] Chinnapaka S., Yang K.S., Surucu Y., Bengur F.B., Arellano J.A., Tirmizi Z., Malekzadeh H., Epperly M.W., Hou W., Greenberger J.S. (2023). Human adipose ECM alleviates radiation-induced skin fibrosis via endothelial cell-mediated M2 macrophage polarization. iScience.

[92] DeCostanza L., Grogan G.M., Bruce A.C., Peachey C.M., Clark E.A., Atkins K., Tylek T., Solga M.D., Spiller K.L., Peirce S.M. (2025). Decellularized porcine dermal hydrogel enhances implant-based wound healing in the setting of irradiation. Acta Biomater..

[93] Chen X., Zhai D., Wang B., Hao S., Song J., Peng Z. (2020). Hair keratin promotes wound healing in rats with combined radiation-wound injury. J. Mater. Sci. Mater. Med..

[94] Johnson M.B., Pang B., Gardner D.J., Niknam-Benia S., Soundarajan V., Bramos A., Perrault D.P., Banks K., Lee G.K., Baker R.Y. (2017). Topical Fibronectin Improves Wound Healing of Irradiated Skin. Sci. Rep..

[95] Xie X.T., Gao C.H., Tan L.F., Chen L.X., Fan J.X., Xiong W., Cheng K., Zhao Y.D., Liu B. (2025). Gene-engineered polypeptide hydrogels with on-demand oxygenation and ECM-cell interaction mimicry for diabetic wound healing. Biomaterials.

[96] Schultze-Mosgau S., Blaese M.A., Grabenbauer G., Wehrhan F., Kopp J., Amann K., Rodemann H.P., Rödel F. (2004). Smad-3 and Smad-7 expression following anti-transforming growth factor beta 1 (TGFbeta1)-treatment in irradiated rat tissue. Radiother. Oncol..

[97] Gallet P., Phulpin B., Merlin J.L., Leroux A., Bravetti P., Mecellem H., Tran N., Dolivet G. (2011). Long-term alterations of cytokines and growth factors expression in irradiated tissues and relation with histological severity scoring. PLoS ONE.

[98] Maria O.M., Eliopoulos N., Muanza T. (2017). Radiation-Induced Oral Mucositis. Front. Oncol..

[99] Peng Y., Jiang Z., Xu S., He L., Jiang T., Yang Y., Xie X., Lei L. (2026). Designing adhesive hydrogels for oral diseases treatment. Mater. Today Bio.

[100] Mndlovu H., du Toit L.C., Kumar P., Choonara Y.E., Marimuthu T., Kondiah P.P.D., Pillay V. (2020). Bioplatform Fabrication Approaches Affecting Chitosan-Based Interpolymer Complex Properties and Performance as Wound Dressings. Molecules.

[101] Wang Z., Han X., Xiao W., Wang P., Wang J., Zou D., Luo X., Shi L., Wu J., Guo L. (2024). Mussel-inspired adhesive drug-loaded hydrogels for oral ulcers treatment. Acta Biomater..

[102] Zhang Q., Liu D., Gao J., Wu X., Hu W., Han L. (2025). Surface-engineered hydrophobic hydrogels via cholesterol micelle rearrangement for robust wet adhesion and oral mucositis therapy. Mater. Today Bio.

[103] Yuan R., Du S., Pan S., Lin Z., Zhang N., Zhang C., Zeng Q., Wei Y., Wu Y., Tao L. (2025). Multifunctional hydrogel encapsulated with baicalin for full-layer regeneration of drug-resistant bacteria-infected wounds after radiotherapy. Bioact. Mater..

[104] Ding Z., Hu X., Liang W., Zheng S., Luo X., Zhao H. (2026). Correction: Dual-functional guanosine-based hydrogel: High-efficiency protection in radiation-induced oral mucositis. J. Mater. Chem. B.

[105] Guo J., Zhang X., Mao R., Li H., Hao Y., Zhang J., Wang W., Zhang Y., Liu J. (2024). Multifunctional Glycopeptide-Based Hydrogel via Dual-Modulation for the Prevention and Repair of Radiation-Induced Skin Injury. ACS Biomater. Sci. Eng..

[106] Wu Y., Jiang L., Li K., Liu J., Chen P., Xu J., Zhang J. (2025). Hyaluronic acid-based composite hydrogels embedded with core-shell microgels with properties of mucosal adhesion and combined drug administration for chemoradiotherapy induced oral mucositis. Int. J. Biol. Macromol..

[107] Moussa L., Pattappa G., Doix B., Benselama S.L., Demarquay C., Benderitter M., Sémont A., Tamarat R., Guicheux J., Weiss P. (2017). A biomaterial-assisted mesenchymal stromal cell therapy alleviates colonic radiation-induced damage. Biomaterials.

[108] Mathieu E., Lamirault G., Toquet C., Lhommet P., Rederstorff E., Sourice S., Biteau K., Hulin P., Forest V., Weiss P. (2012). Intramyocardial delivery of mesenchymal stem cell-seeded hydrogel preserves cardiac function and attenuates ventricular remodeling after myocardial infarction. PLoS ONE.

[109] Kim I.G., Cho H., Shin J., Cho J.H., Cho S.W., Chung E.J. (2021). Regeneration of irradiation-damaged esophagus by local delivery of mesenchymal stem-cell spheroids encapsulated in a hyaluronic-acid-based hydrogel. Biomater. Sci..

[110] Kim W.H., Yoo J.H., Yoo I.K., Kwon C.I., Hong S.P. (2023). Effects of Mesenchymal Stem Cells Treatment on Radiation-Induced Proctitis in Rats. Yonsei Med. J..

[111] Dunn C.M., Kameishi S., Grainger D.W., Okano T. (2021). Strategies to address mesenchymal stem/stromal cell heterogeneity in immunomodulatory profiles to improve cell-based therapies. Acta Biomater..

[112] Han Q., Ai S., Hong Q., Zhang C., Song Y., Wang X., Wang X., Cui S., Li Z., Zhu H. (2022). A supramolecular hydrogel based on the combination of YIGSR and RGD enhances mesenchymal stem cells paracrine function via integrin α2β1 and PI3K/AKT signaling pathway for acute kidney injury therapy. Chem. Eng. J..

[113] Chen G., Han Y., Zhang H., Tu W., Zhang S. (2021). Radiotherapy-Induced Digestive Injury: Diagnosis, Treatment and Mechanisms. Front. Oncol..

[114] Sharma R., Lewis S., Wlodarski M.W. (2020). DNA Repair Syndromes and Cancer: Insights Into Genetics and Phenotype Patterns. Front. Pediatr..

[115] Moussa L., Usunier B., Demarquay C., Benderitter M., Tamarat R., Sémont A., Mathieu N. (2016). Bowel Radiation Injury: Complexity of the Pathophysiology and Promises of Cell and Tissue Engineering. Cell Transplant..

[116] Usunier B., Benderitter M., Tamarat R., Chapel A. (2014). Management of fibrosis: The mesenchymal stromal cells breakthrough. Stem Cells Int..

[117] Rao A.D., Coquia S., Jong R., Gourin C., Page B., Latronico D., Dah S., Su L., Clarke S., Schultz J. (2018). Effects of Biodegradable Hydrogel Spacer Injection on Contralateral Submandibular Gland Sparing in Radiotherapy for Head and Neck Cancers. Radiother. Oncol..

[118] Rucinski A., Brons S., Richter D., Habl G., Debus J., Bert C., Haberer T., Jäkel O. (2015). Ion Therapy of Prostate Cancer: Daily Rectal Dose Reduction by Application of Spacer Gel. Radiat. Oncol..

[119] van Wijk Y., Vanneste B.G.L., Walsh S., van der Meer S., Ramaekers B., van Elmpt W., Pinkawa M., Lambin P. (2017). Development of a Virtual Spacer to Support the Decision for the Placement of an Implantable Rectum Spacer for Prostate Cancer Radiotherapy: Comparison of Dose, Toxicity and Cost-Effectiveness. Radiother. Oncol..

[120] Rao A.D., Feng Z., Shin E.J., He J., Waters K.M., Coquia S., DeJong R., Rosati L.M., Su L., Li D. (2017). A Novel Absorbable Radiopaque Hydrogel Spacer to Separate the Head of the Pancreas and Duodenum in Radiation Therapy for Pancreatic Cancer. Int. J. Radiat. Oncol. Biol. Phys..

[121] Pinkawa M., Berneking V., Schlenter M., Krenkel B., Eble M.J. (2017). Quality of Life After Radiation Therapy for Prostate Cancer with a Hydrogel Spacer: 5-Year Results. Int. J. Radiat. Oncol. Biol. Phys..

[122] Chao M., Ho H., Chan Y., Tan A., Pham T., Bolton D., Troy A., Temelcos C., Sengupta S., McMillan K. (2018). Prospective Analysis of Hydrogel Spacer for Patients with Prostate Cancer Undergoing Radiotherapy. BJU Int..

[123] Armstrong N., Bahl A., Pinkawa M., Ryder S., Ahmadu C., Ross J., Bhattacharyya S., Woodward E., Battaglia S., Binns J. (2021). SpaceOAR Hydrogel Spacer for Reducing Radiation Toxicity During Radiotherapy for Prostate Cancer. A Systematic Review. Urology.

[124] van Gysen K., Kneebone A., Alfieri F., Guo L., Eade T. (2014). Feasibility of and rectal dosimetry improvement with the use of SpaceOAR^®^ hydrogel for dose-escalated prostate cancer radiotherapy. J. Med. Imaging Radiat. Oncol..

[125] Uhl M., Herfarth K., Eble M.J., Pinkawa M., van Triest B., Kalisvaart R., Weber D.C., Miralbell R., Song D.Y., DeWeese T.L. (2014). Absorbable hydrogel spacer use in men undergoing prostate cancer radiotherapy: 12 month toxicity and proctoscopy results of a prospective multicenter phase II trial. Radiat. Oncol..

[126] Afkhami Ardekani M., Ghaffari H. (2020). Optimization of prostate brachytherapy techniques with polyethylene glycol-based hydrogel spacers: A systematic review. Brachytherapy.

[127] Kundu P., Lin E.Y., Yoon S.M., Parikh N.R., Ruan D., Kishan A.U., Lee A., Steinberg M.L., Chang A.J. (2022). Rectal Radiation Dose and Clinical Outcomes in Prostate Cancer Patients Treated with Stereotactic Body Radiation Therapy with and Without Hydrogel. Front. Oncol..

[128] Sadeghi M.H., Siavashpour Z., Sina S. (2025). Tissue spacers in brachytherapy: A systematic review and meta-analysis. Crit. Rev. Oncol. Hematol..

[129] Folkert M.R., Zelefsky M.J., Hannan R., Desai N.B., Lotan Y., Laine A.M., Kim D.W.N., Neufeld S.H., Hornberger B., Kollmeier M.A. (2021). A Multi-Institutional Phase 2 Trial of High-Dose SAbR for Prostate Cancer Using Rectal Spacer. Int. J. Radiat. Oncol. Biol. Phys..

[130] Mahal B.A., O’Leary M.P., Nguyen P.L. (2014). Hydrogel Spacing for Radiotherapy of Prostate Cancer: A Review of the Literature. Urol. Pract..

[131] Hatiboglu G., Pinkawa M., Vallée J.P., Hadaschik B., Hohenfellner M. (2012). Application technique: Placement of a prostate-rectum spacer in men undergoing prostate radiation therapy. BJU Int..

[132] Pinkawa M., Piroth M.D., Holy R., Escobar-Corral N., Caffaro M., Djukic V., Klotz J., Eble M.J. (2013). Spacer stability and prostate position variability during radiotherapy for prostate cancer applying a hydrogel to protect the rectal wall. Radiother. Oncol..

[133] Kim S.H., Ding K., Rao A., He J., Bhutani M.S., Herman J.M., Narang A., Shin E.J. (2021). EUS-guided hydrogel microparticle injection in a cadaveric model. J. Appl. Clin. Med. Phys..

[134] Wu Y.H., Shen S.H., Wang Y.P., Chang N.W., Lee P.C., Li C.P., Lan K.L., Shiau C.Y., Hu Y.W., Huang P.I. (2024). Feasibility estimation of injected hydrodissection before definitive radiotherapy of pancreatic adenocarcinoma. J. Chin. Med. Assoc..

[135] Cirillo G., Spizzirri U.G., Curcio M., Nicoletta F.P., Iemma F. (2019). Injectable Hydrogels for Cancer Therapy Over the Last Decade. Pharmaceutics.

[136] Pang L., Tian P., Cui X., Wu X., Zhao X., Wang H., Wang D., Pan H. (2021). In Situ Photo-Cross-Linking Hydrogel Accelerates Diabetic Wound Healing Through Restored Hypoxia-Inducible Factor 1-Alpha Pathway and Regulated Inflammation. ACS Appl. Mater. Interfaces.

[137] Pandey M., Choudhury H., D’OSegar Singh S.K., Chetty Annan N., Bhattamisra S.K., Gorain B., Mohd Amin M.C.I. (2021). Budesonide-Loaded Pectin/Polyacrylamide Hydrogel for Sustained Delivery: Fabrication, Characterization and In Vitro Release Kinetics. Molecules.

[138] Qiao Y., Zhang Q., Wang Q., Li Y., Wang L. (2021). Filament-Anchored Hydrogel Layer on Polypropylene Hernia Mesh with Robust Anti-Inflammatory Effects. Acta Biomater..

[139] Machado V.S., Camponogara C., Oliveira S.M., Baldissera M.D., Sagrillo M.R., Gundel S.D.S., Silva A.P.T.D., Ourique A.F., Klein B., Wagner R. (2020). Topical Hydrogel Containing Achyrocline Satureioides Oily Extract (Free and Nanocapsule) has Anti-Inflammatory Effects and Thereby Minimizes Irritant Contact Dermatitis. Acad. Bras. Cienc..

[140] Wang Q.-S., Xu B.-X., Fan K.-J., Li Y.-W., Wu J., Wang T.-Y. (2020). Dexamethasone-Loaded Thermosensitive Hydrogel Suppresses Inflammation and Pain in Collagen-Induced Arthritis Rats. Drug Des. Devel. Ther..

[141] Kerdsirichairat T., Narang A.K., Thompson E., Kim S.H., Rao A., Ding K., Shin E.J. (2019). Feasibility of Using Hydrogel Spacers for Borderline-Resectable and Locally Advanced Pancreatic Tumors. Gastroenterology.

[142] Sémont A., François S., Mouiseddine M., François A., Saché A., Frick J., Thierry D., Chapel A. (2006). Mesenchymal stem cells increase self-renewal of small intestinal epithelium and accelerate structural recovery after radiation injury. Adv. Exp. Med. Biol..

[143] Slaughter B.V., Khurshid S.S., Fisher O.Z., Khademhosseini A., Peppas N.A. (2009). Hydrogels in regenerative medicine. Adv. Mater..

[144] Moussa L., Demarquay C., Réthoré G., Benadjaoud M.A., Siñeriz F., Pattapa G., Guicheux J., Weiss P., Barritault D., Mathieu N. (2019). Heparan Sulfate Mimetics: A New Way to Optimize Therapeutic Effects of Hydrogel-Embedded Mesenchymal Stromal Cells in Colonic Radiation-Induced Damage. Sci. Rep..

[145] Jensen M.M., Jia W., Isaacson K.J., Schults A., Cappello J., Prestwich G.D., Oottamasathien S., Ghandehari H. (2017). Silk-elastinlike protein polymers enhance the efficacy of a therapeutic glycosaminoglycan for prophylactic treatment of radiation-induced proctitis. J. Control. Release.

[146] Rimpy Abhishek Ahuja M. (2017). Evaluation of carboxymethyl moringa gum as nanometric carrier. Carbohydr. Polym..

[147] Abhishek Rimpy Ahuja M. (2018). Moringa gum-g-poly(N-vinyl-2-pyrrolidone)—A potential buccoadhesive polymer. Int. J. Biol. Macromol..

[148] Singh B., Kumar A. (2018). Radiation-induced graft copolymerization of N vinyl imidazole onto moringa gum polysaccharide for making hydrogels for biomedical applications. Int. J. Biol. Macromol..

[149] Cruz-Acuña R., Quirós M., Farkas A.E., Dedhia P.H., Huang S., Siuda D., García-Hernández V., Miller A.J., Spence J.R., Nusrat A. (2017). Synthetic hydrogels for human intestinal organoid generation and colonic wound repair. Nat. Cell Biol..

[150] Gu X., Yu L., Wang X., Yin S., Zheng Y., Zheng Z., Zhang Y., Chen K., Zhang Y., Ding Y. (2025). CS@LGG as a therapeutic biomaterial for acute radiation-induced bowel injury alleviation. Adv. Radiother. Nucl. Med..

[151] Bray F., Laversanne M., Sung H., Ferlay J., Siegel R.L., Soerjomataram I., Jemal A. (2024). Global cancer statistics 2022: GLOBOCAN estimates of incidence and mortality worldwide for 36 cancers in 185 countries. CA Cancer J. Clin..

[152] Zhu H., Chua M.L.K., Chitapanarux I., Kaidar-Person O., Mwaba C., Alghamdi M., Rodríguez Mignola A., Amrogowicz N., Yazici G., Bourhaleb Z. (2024). Global radiotherapy demands and corresponding radiotherapy-professional workforce requirements in 2022 and predicted to 2050: A population-based study. Lancet Glob. Health.

[153] Wang S., Xu D., Xiao L., Liu B., Yuan X. (2025). Radiation-induced lung injury: From mechanism to prognosis and drug therapy. Radiat. Oncol..

[154] Chang S., Lv J., Wang X., Su J., Bian C., Zheng Z., Yu H., Bao J., Xin Y., Jiang X. (2024). Pathogenic mechanisms and latest therapeutic approaches for radiation-induced lung injury: A narrative review. Crit. Rev. Oncol. Hematol..

[155] Hanania A.N., Mainwaring W., Ghebre Y.T., Hanania N.A., Ludwig M. (2019). Radiation-Induced Lung Injury: Assessment and Management. Chest.

[156] Giuranno L., Ient J., De Ruysscher D., Vooijs M.A. (2019). Radiation-Induced Lung Injury (RILI). Front. Oncol..

[157] Maier P., Hartmann L., Wenz F., Herskind C. (2016). Cellular Pathways in Response to Ionizing Radiation and Their Targetability for Tumor Radiosensitization. Int. J. Mol. Sci..

[158] Yan Y., Fu J., Kowalchuk R.O., Wright C.M., Zhang R., Li X., Xu Y. (2022). Exploration of radiation-induced lung injury, from mechanism to treatment: A narrative review. Transl. Lung Cancer Res..

[159] Zhang X., Zhang Z., Huang M., Jin Y., Huang Y., Ji P., Ma Z. (2025). Pathological Mechanisms of Radiation-Induced Lung Injury and Novel Nano-Drug Delivery Therapeutic Strategies. Int. J. Nanomed..

[160] Shi Y., Wang S., Yang R., Wang Z., Zhang W., Liu H., Huang Y. (2022). ROS Promote Hypoxia-Induced Keratinocyte Epithelial-Mesenchymal Transition by Inducing SOX2 Expression and Subsequent Activation of Wnt/β-Catenin. Oxid. Med. Cell. Longev..

[161] Robert S., Gicquel T., Victoni T., Valença S., Barreto E., Bailly-Maître B., Boichot E., Lagente V. (2016). Involvement of matrix metalloproteinases (MMPs) and inflammasome pathway in molecular mechanisms of fibrosis. Biosci. Rep..

[162] Zhao R., Liu C., Luo H., Zhao J., Zhang J., He Y., Li Z., Yang P., Xu L., Wan Y. (2024). Nanoengineered Injectable Hydrogel: An Advanced Radioprotective Barrier with Magnetic Hyperthermia Synergy. ACS Appl. Mater. Interfaces.

[163] Zhou J., Wu P., Sun H., Zhou H., Zhang Y., Xiao Z. (2020). Lung tissue extracellular matrix-derived hydrogels protect against radiation-induced lung injury by suppressing epithelial-mesenchymal transition. J. Cell Physiol..

[164] Akbari Lasboo S., Eslami H., Razavi-Tousi T., Ansari M., Afroozan Bazghaleh A. (2024). The affinity of cellulose nanoparticle toward hydrogel based on chitosan/tragacanth for radiation protection: Study of pulmonary damages on rats. Polym. Sci..

[165] Nikjoo D., van der Zwaan I., Brülls M., Tehler U., Frenning G. (2021). Hyaluronic Acid Hydrogels for Controlled Pulmonary Drug Delivery-A Particle Engineering Approach. Pharmaceutics.

[166] Benxu T., He Y. (2024). Amniotic extracelluar matrix microgels for attenuation of radiation-induced lung injury. Med. Hypoth..

[167] Wang H., Wei J., Zheng Q., Meng L., Xin Y., Yin X., Jiang X. (2019). Radiation-induced heart disease: A review of classification, mechanism and prevention. Int. J. Biol. Sci..

[168] Wang L., Zhao L., Wen D., Guo Y., Wang Z., Li S., Wen M., Liu Y. (2026). Development of an injectable conductive silk sericin hydrogel loaded with exosomes for potential treatment of radiation-induced heart disease. Mater. Chem. Phys..

[169] Abouegylah M., Braunstein L.Z., Alm El-Din M.A., Niemierko A., Salama L., Elebrashi M., Edgington S.K., Remillard K., Napolitano B., Naoum G.E. (2019). Evaluation of radiation-induced cardiac toxicity in breast cancer patients treated with Trastuzumab-based chemotherapy. Breast. Cancer Res. Treat..

[170] Zhao C., Xu S., Yang Y., Shen X., Wang J., Xing S., Yu Z. (2025). Intersection of Cardio-Oncology: An Overview of Radiation-Induced Heart Disease in the Context of Tumors. J. Am. Heart Assoc..

[171] Umezawa R., Takase K., Jingu K., Takanami K., Ota H., Kaneta T., Takeda K., Matsushita H., Ariga H., Takahashi S. (2013). Evaluation of radiation-induced myocardial damage using iodine-123 β-methyl-iodophenyl pentadecanoic acid scintigraphy. J. Radiat. Res..

[172] Hao T., Li J., Yao F., Dong D., Wang Y., Yang B., Wang C. (2017). Injectable fullerenol/alginate hydrogel for suppression of oxidative stress damage in brown adipose-derived stem cells and cardiac repair. ACS Nano.

[173] Zhou J., Liu W., Zhao X., Xian Y., Wu W., Zhang X., Zhao N., Xu F.J., Wang C. (2021). Natural Melanin/Alginate Hydrogels Achieve Cardiac Repair through ROS Scavenging and Macrophage Polarization. Adv. Sci..

[174] Zhang L., Bei Z., Li T., Qian Z. (2023). An injectable conductive hydrogel with dual responsive release of rosmarinic acid improves cardiac function and promotes repair after myocardial infarction. Bioact. Mater..

[175] Guan H., Liu J., Liu D., Ding C., Zhan J., Hu X., Zhang P., Wang L., Lan Q., Qiu X. (2022). Elastic and conductive melanin/poly(vinyl alcohol) composite hydrogel for enhancing repair effect on myocardial infarction. Macromol. Biosci..

[176] Hu C., Liu W., Long L., Wang Z., Zhang W., He S., Lu L., Fan H., Yang L., Wang Y. (2022). Regeneration of infarcted hearts by myocardial infarction-responsive injectable hydrogels with combined anti-apoptosis, anti-inflammatory and pro-angiogenesis properties. Biomaterials.

[177] Wang X., Shi H., Huang S., Zhang Y., He X., Long Q., Qian B., Zhong Y., Qi Z., Zhao Q. (2023). Localized delivery of anti-inflammatory agents using extracellular matrix-nanostructured lipid carriers hydrogel promotes cardiac repair post-myocardial infarction. Biomaterials.

[178] McLaughlin S., McNeill B., Podrebarac J., Hosoyama K., Sedlakova V., Cron G., Smyth D., Seymour R., Goel K., Liang W. (2019). Injectable human recombinant collagen matrices limit adverse remodeling and improve cardiac function after myocardial infarction. Nat. Commun..

[179] Lyu Y., Xie J., Liu Y., Xiao M., Li Y., Yang J., Yang J., Liu W. (2020). Injectable hyaluronic acid hydrogel loaded with functionalized human mesenchymal stem cell aggregates for repairing infarcted myocardium. ACS Biomater. Sci. Eng..

[180] Han C., Zhou J., Liang C., Liu B., Pan X., Zhang Y., Wang Y., Yan B., Xie W., Liu F. (2019). Human umbilical cord mesenchymal stem cell derived exosomes encapsulated in functional peptide hydrogels promote cardiac repair. Biomater. Sci..

[181] Cui Z., Ni N.C., Wu J., Du G.-Q., He S., Yau T.M., Weisel R.D., Sung H.-W., Li R.-K. (2018). Polypyrrole-chitosan conductive biomaterial synchronizes cardiomyocyte contraction and improves myocardial electrical impulse propagation. Theranostics.

[182] Chen J., Han X., Deng J., Zhang J., Li L., Ni J., Huang Y., Xie X., Chen S., Ke L. (2021). An injectable hydrogel based on phenylboronic acid hyperbranched macromer encapsulating gold nanorods and astragaloside IV nanodrug for myocardial infarction. Chem. Eng. J..

[183] Zhou J., Yang X., Liu W., Wang C., Shen Y., Zhang F., Zhu H., Sun H., Chen J., Lam J. (2018). Injectable OPF/graphene oxide hydrogels provide mechanical support and enhance cell electrical signaling after implantation into myocardial infarct. Theranostics.

[184] Zhu S., Yu C., Liu N., Zhao M., Chen Z., Liu J., Li G., Huang H., Guo H., Sun T. (2022). Injectable conductive gelatin methacrylate/oxidized dextran hydrogel encapsulating umbilical cord mesenchymal stem cells for myocardial infarction treatment. Bioact. Mater..

[185] Ke X., Li M., Wang X., Liang J., Wang X., Wu S., Long M., Hu C. (2020). An injectable chitosan/dextran/β -glycerophosphate hydrogel as cell delivery carrier for therapy of myocardial infarction. Carbohydr. Polym..

[186] Melly L., Grosso A., Stanciu Pop C., Chu Y.-H. (2020). Fibrin hydrogels promote scar formation and prevent therapeutic angiogenesis in the heart. J. Tissue Eng. Regen. Med..

[187] Tarsitano M., Liu Chung Ming C., Idais D., Mahmodi H., Wyllie K., Isella B., Cox T.R., Kabakova I., Paolino D., Gentile C. (2025). Sericin improves alginate-gelatin hydrogels’ mechanical properties, porosity, durability, and viability of fibroblasts in cardiac spheroids. Int. J. Bioprint..

[188] Pu X., Ma S., Gao Y., Xu T., Chang P., Dong L. (2020). Mesenchymal Stem Cell-Derived Exosomes: Biological Function and Their Therapeutic Potential in Radiation Damage. Cells.

[189] Liu R., Bian Y., Liu L., Liu L., Liu X., Ma S. (2022). Molecular pathways associated with oxidative stress and their potential applications in radiotherapy (Review). Int. J. Mol. Med..

[190] Li Y., Zhou Z., Xu S., Jiang J., Xiao J. (2023). Review of the Pathogenesis, Diagnosis, and Management of Osteoradionecrosis of the Femoral Head. Med. Sci. Monit..

[191] Zhai J., He F., Wang J., Chen J., Tong L., Zhu G. (2019). Influence of radiation exposure pattern on the bone injury and osteoclastogenesis in a rat model. Int. J. Mol. Med..

[192] Delanian S., Lefaix J.L. (2004). The radiation-induced fibroatrophic process: Therapeutic perspective via the antioxidant pathway. Radiother. Oncol..

[193] Zhao W., Robbins M.E. (2009). Inflammation and chronic oxidative stress in radiation-induced late normal tissue injury: Therapeutic implications. Curr. Med. Chem..

[194] Wang Y., Turkstani H., Alfaifi A., Akintoye S.O. (2024). Diagnostic and Therapeutic Approaches to Jaw Osteoradionecrosis. Diagnostics.

[195] Topkan E., Kucuk A., Somay E., Yilmaz B., Pehlivan B., Selek U. (2023). Review of Osteoradionecrosis of the Jaw: Radiotherapy Modality, Technique, and Dose as Risk Factors. J. Clin. Med..

[196] Chronopoulos A., Zarra T., Tröltzsch M., Mahaini S., Ehrenfeld M., Otto S. (2015). Osteoradionecrosis of the mandible: A ten year single-center retrospective study. J. Craniomaxillofac. Surg..

[197] Liu L., Luo P., Wen P., Xu P. (2024). The role of magnesium in the pathogenesis of osteoporosis. Front. Endocrinol..

[198] Wang Q., Hu X., Xiao Z., Ye K., Li J., Tan J., Rao N., Zhang D., Sun G., Cai M. (2025). Magnesium ion hydrogel enhances resistance to radiation-induced bone injury by modulating the bone immune microenvironment and promoting microvascularization. Regen. Biomater..

[199] Li L., Gu Z., Wu Y., Lin S., Cheng X., Zhang L.W., Qin J., Dong Q., Wang Y., Wang Y. (2025). Hyaluronic acid and polyvinyl alcohol-based radioresponsive hydrogel for combined radioimmunotherapy of breast cancer bone metastasis. Carbohydr. Polym..

[200] Zhao T., Chen L., Yu C., He G., Lin H., Sang H., Chen Z., Hong Y., Sui W., Zhao J. (2024). Effect of injectable calcium alginate–amelogenin hydrogel on macrophage polarization and promotion of jawbone osteogenesis. RSC Adv..

[201] Lv N., Zhou Z., Hong L., Li H., Liu M., Qian Z. (2024). Zinc-energized dynamic hydrogel accelerates bone regeneration via potentiating the coupling of angiogenesis and osteogenesis. Front. Bioeng. Biotechnol..

[202] Dong S., An S., Saiding Q., Chen Q., Liu B., Kong N., Chen W., Tao W. (2025). Therapeutic Hydrogels: Properties and Biomedical Applications. Chem. Rev..

[203] Correa S., Grosskopf A.K., Lopez Hernandez H., Chan D., Yu A.C., Stapleton L.M., Appel E.A. (2021). Translational Applications of Hydrogels. Chem. Rev..

[204] Galante R., Pinto T.J.A., Colaço R., Serro A.P. (2018). Sterilization of hydrogels for biomedical applications: A review. J. Biomed. Mater. Res. B Appl. Biomater..

[205] Tavakoli S., Klar A.S. (2020). Advanced Hydrogels as Wound Dressings. Biomolecules.

[206] Vasile C., Pamfil D., Stoleru E., Baican M. (2020). New Developments in Medical Applications of Hybrid Hydrogels Containing Natural Polymers. Molecules.

